# Evaluating the Hypoxia Response of Ruffe and Flounder Gills by a Combined Proteome and Transcriptome Approach

**DOI:** 10.1371/journal.pone.0135911

**Published:** 2015-08-14

**Authors:** Jessica Tiedke, Janus Borner, Hendrik Beeck, Marcel Kwiatkowski, Hanno Schmidt, Ralf Thiel, Andrej Fabrizius, Thorsten Burmester

**Affiliations:** 1 Institute of Zoology, University of Hamburg, Hamburg, Germany; 2 Department of Clinical Chemistry, University Medical Centre Hamburg-Eppendorf, Campus Forschung, Hamburg, Germany; 3 Institute of Molecular Genetics, Johannes Gutenberg-University Mainz, Mainz, Germany; 4 Zoological Museum, Centre of Natural History, University of Hamburg, Hamburg, Germany; Queen's University Belfast, UNITED KINGDOM

## Abstract

Hypoxia has gained ecological importance during the last decades, and it is the most dramatically increasing environmental factor in coastal areas and estuaries. The gills of fish are the prime target of hypoxia and other stresses. Here we have studied the impact of the exposure to hypoxia (1.5 mg O_2_/l for 48 h) on the protein expression of the gills of two estuarine fish species, the ruffe (*Gymnocephalus cernua*) and the European flounder (*Platichthys flesus*). First, we obtained the transcriptomes of mixed tissues (gills, heart and brain) from both species by Illumina next-generation sequencing. Then, the gill proteomes were investigated using two-dimensional gel electrophoresis and mass spectrometry. Quantification of the normalized proteome maps resulted in a total of 148 spots in the ruffe, of which 28 (18.8%) were significantly regulated (> 1.5-fold). In the flounder, 121 spots were found, of which 27 (22.3%) proteins were significantly regulated. The transcriptomes were used for the identification of these proteins, which was successful for 15 proteins of the ruffe and 14 of the flounder. The ruffe transcriptome dataset comprised 87,169,850 reads, resulting in an assembly of 72,108 contigs (N50 = 1,828 bp). 20,860 contigs (26.93%) had blastx hits with E < 1e-5 in the human sequences in the RefSeq database, representing 14,771 unique accession numbers. The flounder transcriptome with 78,943,030 reads assembled into 49,241 contigs (N50 = 2,106 bp). 20,127 contigs (40.87%) had a hit with human proteins, corresponding to 14,455 unique accession numbers. The regulation of selected genes was confirmed by quantitative real-time RT-PCR. Most of the regulated proteins that were identified by this approach function in the energy metabolism, while others are involved in the immune response, cell signalling and the cytoskeleton.

## Introduction

An "estuary" can be defined as a semi-enclosed water body where freshwater from rivers mingles with salt water from oceans [1]. For fish populations, estuaries are essential feeding grounds, nursery areas, spawning places and migration routes [2]. During the last decades, estuaries have been increasingly suffering from degradation caused by changes in temperature and oxygen availability, as well as anthropogenic influences such as the input of nutrients and morphological changes [3, 4]. One of the key issues is the dramatically increasing frequency of hypoxic periods in coastal areas and estuaries [5–9]. In aquatic ecosystems, hypoxia typically refers to a reduced PO_2_ below 2 mg of O_2_ per litre, which is a crucial value for the survival of numerous aquatic species [6, 10]. The oxygen saturation in estuaries is mainly affected by seasonal temperature increases and eutrophication. Hypoxia may additionally lead to the reduction of habitats for fish populations by making deeper, cooler water unavailable in the summer and by interrupting migration routes [6, 10].

Typically, fish respond to hypoxia by a number of behavioural, morphological, physiological and molecular changes. For example, fishes reduce their energy demand by reducing their swimming activity and by active avoidance of hypoxic areas [11, 12]. In response to long-term hypoxia, cyprinid fishes may remodel their gills to increase the respiratory surface [11, 13, 14]. The levels of respiratory proteins (hemoglobin, myoglobin and neuroglobin) may rise in response to hypoxia, and the O_2_ uptake by the blood may be enhanced by an increase of the O_2_ affinity of hemoglobin [15–17]. Hypoxia also induces the expression of genes that code for enzymes of the glycolytic pathway and fermentation, while genes that are involved in aerobic energy production and energy-consuming processes are repressed [18–20].

Several studies have focused on the molecular response to hypoxia in model organisms (e.g. [18, 21–24]), but the knowledge of the impact of hypoxia on fish species that are naturally exposed to hypoxia in their environment is limited [25]. Here we studied the hypoxia response of two estuarine fish species, the European flounder (*Platichthys flesus*) and the ruffe (*Gymnocephalus cernua*). The European flounder is a demersal fish species that mainly resides in estuaries, with the exception of spawning periods [26, 27]. Flounders are known to cope with low oxygen availability by maintaining adequate oxygen extraction rates [28, 29]. Like the flounder, the ruffe is most abundant in estuaries [30]. Ruffes are rather undemanding concerning their spawning grounds or prey and thus can adapt to even degraded environments. Their good adaptability makes them a successful invasive species in Northern America, where they pose a threat to native fish species [31, 32].

In a previous study, we employed quantitative real-time RT-PCR to study the response of different tissues from the ruffe and the European flounder to hypoxia [25]. We compared specimens from the field and the laboratory, and showed that the hypoxia-response in the laboratory largely corresponds to that in the field. Here we specifically focussed on the molecular response to hypoxia in the gills. The gills were chosen because they are the main site of gas exchange in fish, and, therefore, the first target in a hypoxic event [25, 33] and under environmental stresses [34]. We employed a combination of proteome and transcriptome methods, which allowed the easy identification of proteins even in non-model species. This is the first global analysis of transcript and protein abundances in European flounder as well as in ruffe, providing biological insights into the mechanisms of adaption to environmental hypoxia of key estuarine fish species as well as a database for further studies in these non-model fish species.

## Material and Methods

### Ethics statement

Adult ruffes (*G*. *cernua*) (15–20 cm) and European flounders (*P*. *flesus*) (23–28 cm) were collected by a commercial fishing vessel in the Elbe estuary near the city of Glückstadt (near river km 670), Germany, in October 2010. No permit was required for commercial fishing. Both species are neither endangered nor protected. All animal care and experimental procedures of this study were approved by the Hamburg authorities (Behörde für Soziales, Familie, Gesundheit und Verbraucherschutz) under the license no. 04/10.

### Hypoxia treatment

For the initial acclimation, fishes caught in the estuary were placed in a separate box and tap water was added to the estuarine water with a flow rate of about three drops per second until salinity reached < 1%. The fishes were further acclimated to laboratory conditions in a 750-litre aquarium at 11°C with a day/night light cycle of 11.5/12.5 h for four weeks. During acclimation, fish were fed daily with common earthworm (*Lumbricus terrestris*). For hypoxia experiments, male specimens of each species were exposed for 48 h to 1.5 ± 0.1 mg/l dissolved O_2_ (DO) at 11°C. Only male individuals were used to minimise the effect of the hormone status due to the reproductive cycle. As controls, the same number of specimens of each species were treated in parallel under the same conditions, except that the oxygen partial pressure was set to 11.0 ± 0.1 mg/l DO. The experiments were carried out in 100-liter tanks. Oxygen partial pressure was adjusted by a Roxy-1 controller (Sable Systems, Las Vegas, USA) connected to a liquid nitrogen source and monitored with the sensor Oxi340i (WTW, Germany). During the experiments, both treatment groups were not fed. Immediately after the treatments, the individuals were anesthetized in ice-cold water to prevent stress and pain right before killing by cutting the spinal cord and the aorta dorsalis. Gills, brains and hearts were excised, washed in cold saline to remove blood contamination and immediately frozen in liquid nitrogen.

### Isolation of RNA

The frozen tissues were ground to a fine powder with liquid nitrogen using a mortar and pestle. The isolation of total RNA was performed with peqGOLD Trifast (peqLAB Biotechnology GmbH, Erlangen, Germany) in combination with an RNeasy Mini Kit, including an on-column digestion with RNase-free DNase (Qiagen, Hilden, Germany). Total RNA was quantified spectrometrically with the Nanodrop ND 100 UV-Vis spectrometer (Thermo Scientific, Bonn, Germany). The integrity of the RNA was checked by formaldehyde agarose gel electrophoresis.

### cDNA library construction and Illumina sequencing

For each species, 3.3 μg RNA of each gill, brain and heart from single (to avoid problems due to multiple alleles) untreated specimens were combined, resulting in 10 μg of total RNA of the mixed tissues. Library construction and Illumina sequencing were performed by MWG Operon (Ebersberg, Germany). Briefly, polyA^+^-RNA was purified using oligo(dT) beads. The cDNA synthesis was carried out using random hexamer primers. From this cDNA-pool, fragments suitable for 100 bp paired-end library sequencing were selected and purified by agarose gel electrophoresis. The sequencing library was generated by PCR amplification and sequencing was performed using the HiSeq 2000 platform (Illumina). The quality of the raw sequence data was checked with FastQC (http://www.bioinformatics.babraham.ac.uk/projects/fastqc/). The raw files are available from the NCBI SRA database under the accession numbers SRR1822420 and SRR1822477 (Bioprojects PRJNA276806 and PRJNA276828).

### Sequence assembly and functional annotation

The raw Illumina reads were processed with the aid of the FASTX-Toolkit (http://hannonlab.cshl.edu/fastx_toolkit/) by trimming the adaptor sequences (the first 14 nucleotides) and removing low quality reads (cut-off q20). The sequences were assembled employing the CLC Genomics Workbench 7.5 (CLC Bio). For annotation, the assembled contigs were run using the CLC local-blastx against the human (build38) and zebrafish (build10) non-redundant RefSeq protein-databases. Only hits with an E-value of E ≤ 1e-5 were treated as significant. Gene Ontology annotation was performed using the PANTHER online tool [35] by submitting the RefSeq-IDs retrieved from the blastx-searches and choosing the appropriate organism for annotation. To assess the overlap of the different assemblies, local blastn searches were conducted using the ruffe contigs against a database with the flounder contigs and vice versa. To visualise the overlap we extracted all blast hits resulting in the same contig pair used as query (flounder or ruffe) and producing the same hit in the appropriate database (flounder or ruffe) with an e-value cut-off ≤ 1e-10.

### Two-dimensional gel electrophoresis and gel image analyses

For quantitative protein analyses, we used whole gills from at three specimens from both treatment groups (hypoxia and normoxia) for each species. After homogenization with a mortar and pestle in liquid nitrogen, the tissue powder was sonicated in 150 μl lysis buffer (0.02 M HEPES, pH 7.5), containing the Complete protease inhibitor mix (Roche Diagnostic, Mannheim, Germany). The homogenates were centrifuged, the supernatants were collected and protein concentrations were determined using the BCA method [36].

50 mg protein of each sample were added to rehydration buffer (8 M urea, 2 M thiourea, 0.016 M CHAPS, 0.02 M dithiothreitol, 0.5% (v/v) carrier ampholytes 3–10) to a final volume of 125 μl, and loaded onto a linear Immobiline DryStrip pH 4–7 (13 cm; GE Healthcare, Freiburg, Germany) where they were allowed to rehydrate over night. The isoelectric focussing (IEF) was conducted for 2 h at 300 V, followed by a 3 h gradient at 300 V to 3500 V and 2 h at 3500 V. After IEF the strips equilibrated in a two-buffer system (A and B) for 30 min each. The equilibration buffer was based on a stock solution containing 6 M urea, 30% (v/v) glycerol and 4% (v/v) SDS) in 0.15 M Tris/HCl pH 8.8, which was either supplemented with 0.023 M dithiothreitol (A) or 0.25 M iodoacetamide (B). SDS-PAGE in the second dimension was carried out on 4–15% gradient gel (Biorad, Munich, Germany). After electrophoresis, gels were fixed in 5% (v/v) acetic acid in 1:1 (v/v) water:methanol for 5 h. For the visualisation of protein spots, the blue silver colloidal Coomassie dye protocol was used [37].

For gel image analyses, we used three biological replicates of each species and treatment group. The gel images (with one gel per specimen) were aligned and fused into a master image for each species using DECODON Delta2D software (Version 4.3 Decodon, Greifswald, Germany) according to the manufacturers' specifications. The master images were used for the creation of whole gill proteome maps for each species, which were transferred to every original gel for quantification of the protein spot volumes. The protein expression levels were normalized according to the protein spot that was found to be the least-regulated protein in gel image analyses. Ratios of relative changes in the protein spot volumes were calculated by group-wise comparisons of the hypoxia to the normoxia groups. The significance of differential protein levels was calculated by a Student's t-test with Bonferroni correction of the p-values for multiple testing with a significance level of *p* < 0.05. In addition, significance was calculated with the DECODON Delta2D software on the log2 transformed values of the spot size by a Student's t-test, considering p < 0.1 as significant (this threshold was chosen to accommodate for the variations in natural populations). Protein spots that met both criteria and that had spot volumes that significantly differ at least 1.5-fold were chosen for further identification by mass spectrometry.

### Mass spectrometry and protein identification

Tryptic digestion of the spots from the 2D gel electrophoresis was performed according to Shevchenko et al. [38]. Identification was performed on an Agilent 1100 LC/MSD Trap XCT Ultra equipped with a Chip Cube system using a Large Capacity Chip (II) (Agilent Technologies, Santa Clara, USA). Sample loading onto the enrichment column was performed at a flow rate of 8 μl/min (98% mobile phase A: 0.2% formic acid in H_2_O. 2% mobile phase B: 100% acetonitrile). Tryptic peptides were eluted from the reversed-phase column with a flow rate of 200 nl/min using a linear gradient of 2–40% B in 23 min. MS scans were performed in positive ion mode with a scan range of 300–1500 m/z and a capillary voltage of 1850 V. Flow and temperature of the drying gas were 4 l/min and 350°C. MS/MS scans were carried out in data-dependent acquisition mode (DDA) using the following parameters: scan range: 100–1500 m/z, window for precursor ion selection: 4 Da, fragmentation amplitude: 1.25 V, exclusion time: 0.2 min. MS/MS peak lists were exported as mgf files using Data Analysis Software 6.2 (Agilent Technologies, Santa Clara, USA).

Based on the contigs derived from the ruffe and flounder transcriptomes, all open reading frames longer than 150 bp were translated into an amino acid sequence, from which the species-specific databases were created. These databases were used for protein identification, which was performed with the OpenMS software framework [39] and its graphical user interface TOPPAS (The OpenMS proteomic pipeline [40]) using a consensus identification pipeline with two different search engines (OMSSA [41] and XTandem [42]). The MS/MS data were searched against a protein database containing all sequence data from Actinopterygii in the UniProt Knowledgebase (UniProtKB) and against the species-specific databases that were generated from the trancriptome contigs, using the following parameters: only tryptic peptides with up to one miss cleavage were allowed, 1.2 Da mass tolerances for precursor ions and 0.6 Da for fragment ions, carbamidomethylation on cysteine residues and an oxidation of methionine residues were permitted as variable modifications. Peptides were regarded as correctly identified if the q-value was below 0.01. Additionally, the MS/MS data were searched against the NCBI nr database employing the Mascot MS/MS Ions Search (Matrix Science Ltd, London, UK) with a significance threshold of p < 0.05.

### Quantitative real-time RT-PCR

Quantitative real-time RT-PCR (qRT-PCR) experiments were carried out on an ABI 7500 Fast Real-Time PCR system employing the ABI Power SYBR Green Master Mix (Applied Biosystems, Darmstadt, Germany). For each reaction, the amount of cDNA equivalent to 25 ng total RNA was applied. qRT-PCR was carried out with a standard cycling protocol using 40 amplification cycles (95°C 10 min, 95°C 15 sec, 60°C 30 sec, 72°C 30 sec). Primer sequences and amplicon sizes for each gene are given in S1 table. Success and specificity of the amplification were evaluated using dissociation curves with a temperature range from 60°C to 95°C. Each reaction was performed in triplicate. mRNA copy numbers were calculated using a standard plasmid approach. The first evaluation of the data was done with the ABI 7500 Sequence Detection Software V.2.0.6 (Applied Biosystems). Reaction efficiency was determined by the slope of the standard curve for each gene by analysing a 10-fold dilution of the standard plasmid. For the evaluation of relative expression levels with the 2^-ΔΔCT^ method [43], mean values of all triplicates were used. Further calculations were carried out with the Microsoft Office Excel XP spreadsheet program.

## Results

### Sequencing and characterisation of ruffe and flounder transcriptomes

As a robust background for annotation of differentially expressed proteins, we first obtained the transcriptomes of each ruffe and European flounder using Illumina sequencing. To maximise the gene coverage of this approach, we applied a combination of RNA from functionally distinct organs (gills, brains and hearts) from both species. Illumina sequencing resulted in a total of 87,169,850 read pairs from the ruffe transcriptome and 78,943030 read pairs from the flounder transcriptome (Table 1). Both transcriptomes showed overall high quality, with quality scores of ≥ 30 (base call accuracy of 99.9%) in the raw reads for about 85% of the bases. Assembly was performed with CLC Genomics Workbench, including a re-assembly step to remove redundancy. We obtained 72,108 contigs with an N50 of 1,828 bp (ranging from 53–20,637 bp) for the ruffe transcriptome; the assembly of the flounder transcriptome resulted in 49,241 contigs with an N50 of 2,106 bp (ranging from 53–16,894 bp) (Table 1). 40,547,885 reads (46.52%) mapped back to the assembled transcriptomes in ruffe and 36,667,070 (46.45%) in flounder, respectively.

**Table 1:** Summary of Illumina sequencing and assembly of the transcriptomes.

	Ruffe (*G*. *cernua*)	Flounder (*P*. *flesus*)
Sequences in pairs	87,169,850	78,943,030
Total nucleotides	7,407,977,682	6,695,503,487
Contigs	72,108	49,241
Contigs ≥ 500 bp	34,573	29,991
Max length (bp)	20,637	16,894
Min length (bp)	53	53
N50	1,828	2,106
Nucleotides	65,311,485	58,052,715

The Illumina reads were processed by trimming the adaptor sequences and removing low-quality sequences (cut-off q20). The sequences were assembled employing the CLC Genomics Workbench 7.5 (CLC Bio).

**Fig 1:**
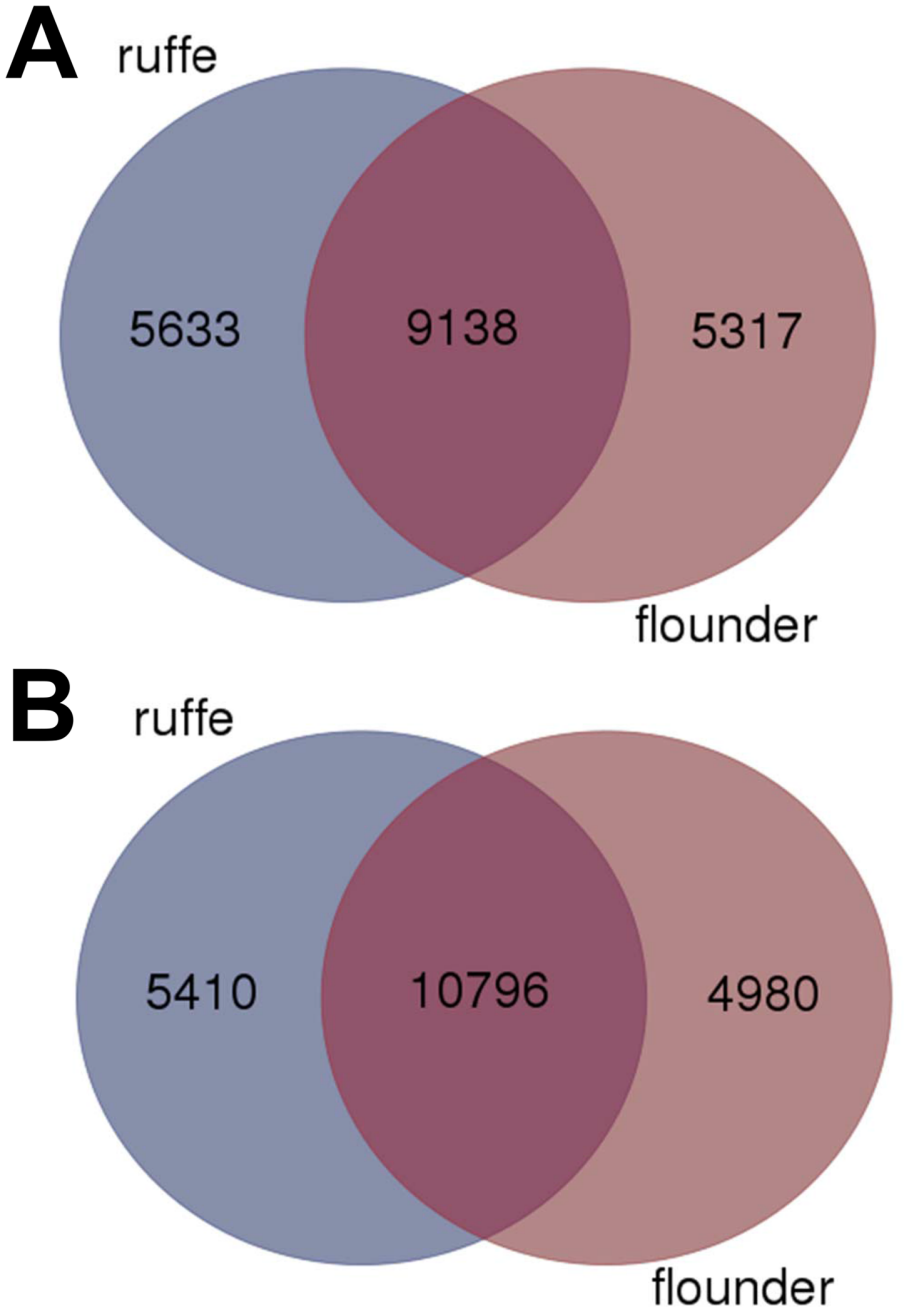
Comparison of ruffe and European flounder transcriptomes. The Venn diagram shows the overlap of the sequences identified in the human (A) and zebrafish (B) RefSeq sequences employing blastx with an E-value cut-off of 1e-5.

For functional annotation, the assembled contigs were compared to the human and zebrafish proteins in the RefSeq database using blastx with an E-value cut-off of 1e-5 (Table 2). In the ruffe transcriptome, a total of 20,860 contigs (26.93%) had blastx hits against the human proteome, representing 14,771 unique accession numbers. When the zebrafish proteome was used, 23,059 contigs (31.98%) were found to have a significant hit with E < 1e-5, which reflect 16,206 unique accession numbers. For the flounder transcriptome, 20,127 contigs (40.87%) had a hit within the human proteins with E < 1e-5, which correspond to 14,455 unique accession numbers. Against the zebrafish proteome for 22,025 contigs (44.73%) hits were found (15,776 unique accession numbers). A comparison of the annotations found in both transcriptomes (i.e., blastx hits) showed an overlap of 9,138 genes, representing 61.9% and 63.2% of the whole transcriptomes of ruffe and flounder, respectively, when the human unique accession numbers (Fig 1A) were used. The same procedure with zebrafish accession numbers resulted in 10,796 shared genes, representing 73.1% and 74.7% of the whole transcriptomes of ruffe and flounder, respectively (Fig 1B).

**Table 2:** Functional annotation of ruffe and flounder transcriptomes.

	Ruffe (*G*. *cernua*)	Flounder (*P*. *flesus*)
BLAST hits (human RefSeq)	20,860	20,127
BLAST hits (human RefSeq) with unique accession numbers	14,771	14,455
BLAST hits (zebrafish RefSeq)	23,059	22,025
BLAST hits (zebrafish RefSeq) with unique accession numbers	16,206	15,776

Functional annotation was done using BLASTx with a cut-off of E < 1e-5.

The ruffe and flounder transcriptomes were compared by performing a reciprocal blastn search, employing the contigs ≥ 500 bp. A total of 17,928 matching pairs of contigs were identified that produced reciprocal best hits. 12,774 contigs of the ruffe produced a different contig in the flounder when used as query; 11,110 contigs of the flounder had a unique hit within the ruffe transcriptome when used as query (S2 Table; S1 Fig). A small number of contigs meaning 40 in ruffe and 24 in flounder, respectively, produced no significant hit (i.e. e-value 1e-10) in the reciprocal blastn approach (S2 Table).

**Fig 2:**
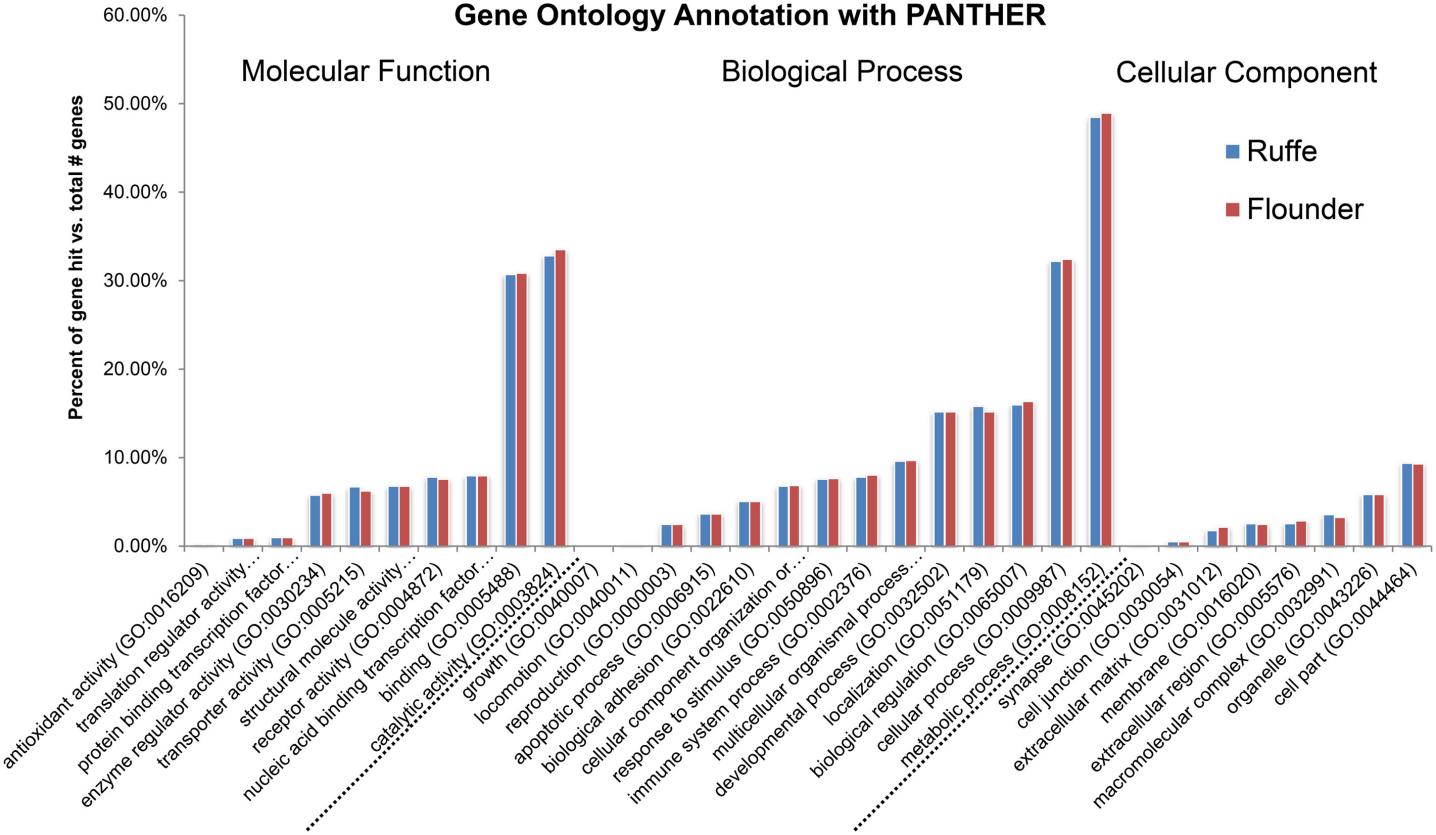
Functional classification and comparison of transcriptomes of the ruffe and European flounder. GO analysis and annotation of the genes were performed with PANTHER [35].

Gene ontology (GO) analyses were conducted on the basis of the contigs that had significant similarities to entries (blastx E < 1e-5) in the human RefSeq protein database, which was chosen because it has a better coverage in GO terms than the zebrafish database. GO annotation according to Blake et al. [44] was applied using the PANTHER classification system [35]. In the ruffe transcriptome, 9,212 contigs (44.2%) had links to at least one GO term. In the flounder transcriptome, 9,112 contigs (45.3%) were linked with at least one GO term. Comparison of the individual GO terms showed a similar pattern in both transcriptomes (Fig 2; S3 Table). Briefly, genes involved in "metabolic process" (GO:0008152) were found highest in both (4470 in ruffe, 4468 in flounder), followed by "catalytic activity" (GO:0003824) (3020/3057), "cellular process" (GO:0009987) (2969/2949) and "binding" (GO:0005488) (2830/2818).

### Effect of hypoxia on the gill proteomes of ruffe and flounder

**Fig 3:**
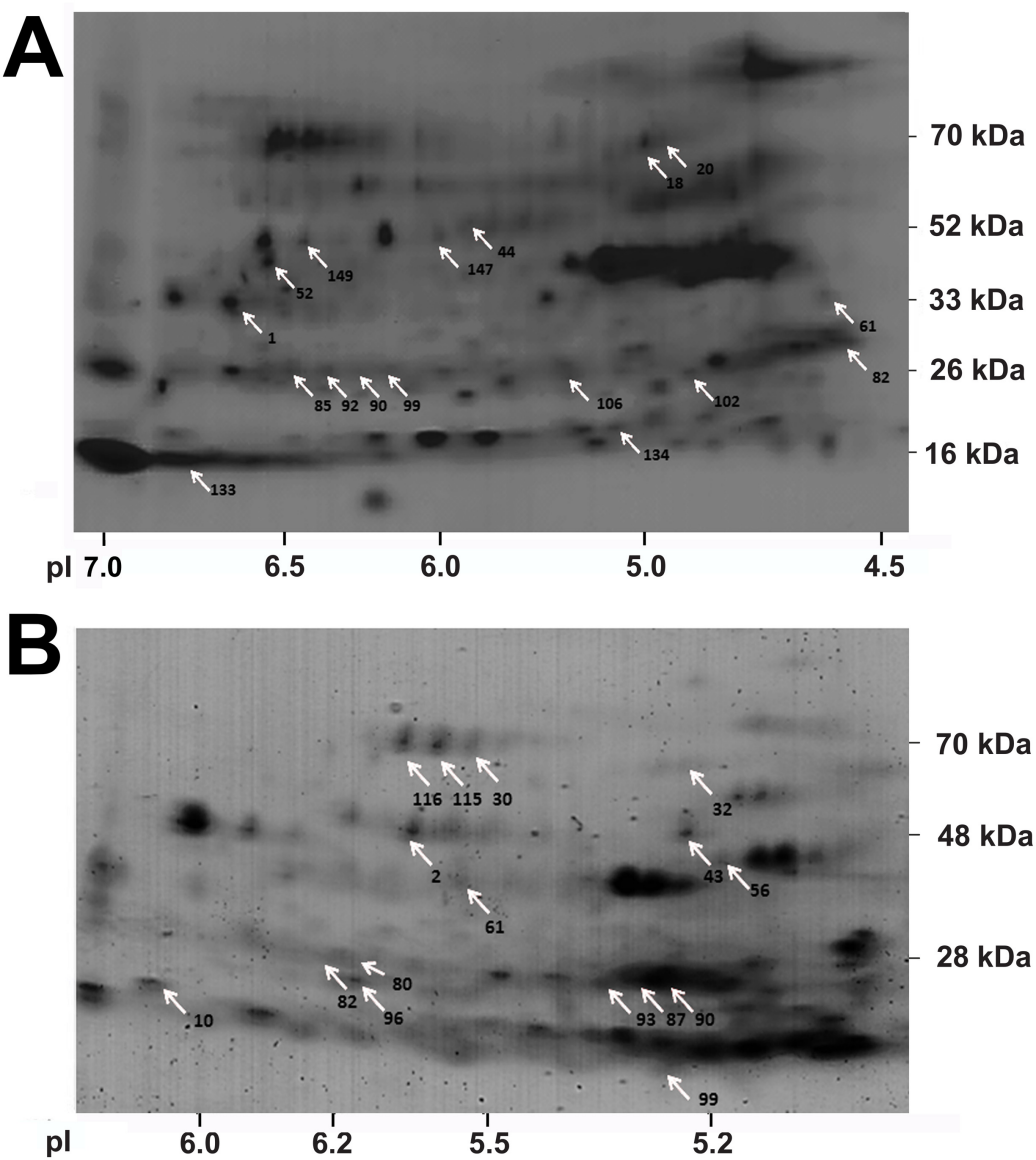
Gill proteome maps. The images were composed of six gel images (three from hypoxia and three from normoxia experiment) of the ruffe (A) and the European flounder (B). Protein spots that significantly changed in abundance in response to hypoxia are labelled with numbers. The original gels are given in S1 Appendix. The lists of spots that changed under hypoxia are given in S4 and S5 Tables.

We investigated the response in the proteomes of the gills of ruffe and flounder to a 48 h exposure hypoxia (1.5 mg/l DO) for 48 h. First, whole proteome maps were created for each species by merging all two-dimensional gels from normoxia (n = 3) and hypoxia (n = 3) (Fig 3). The proteome maps presented at least 149 and 121 well-focused protein spots in the ruffe and the flounder, respectively (Table 3). The spot volumes were normalised according to the different isoforms of GAPDH, which showed the lowest variation in expression levels (Fig 3A, spot 2; Fig 3B, spot 1).

**Table 3:** Evaluation of protein expression profiles in the ruffe and the European flounder.

	Ruffe (*G*. *cernua*)		Flounder (*P*. *flesus*)	
	no. of spots	relative	no. of spots	relative
spots detected	149	100%	121	100%
> 1.5 increase	40	26.8%	24	19.8%
with p- value < 0.1	14	9.4%	6	4.9%
> 1.5 decrease	35	23.5%	49	40.5%
with p- value < 0.1	14	9.4%	21	17.4%

Evaluation of protein expression profiles in the ruffe and the flounder. After spot detection with the proteomics software Delta 2D, proteins with a more than 1.5-fold up/downregulation were identified. The statistical significance of proteins with a more than 1.5-fold up/downregulation was calculated by Student's t-tests on raw and log2 transformed data (see "Material and methods" section).

**Fig 4:**
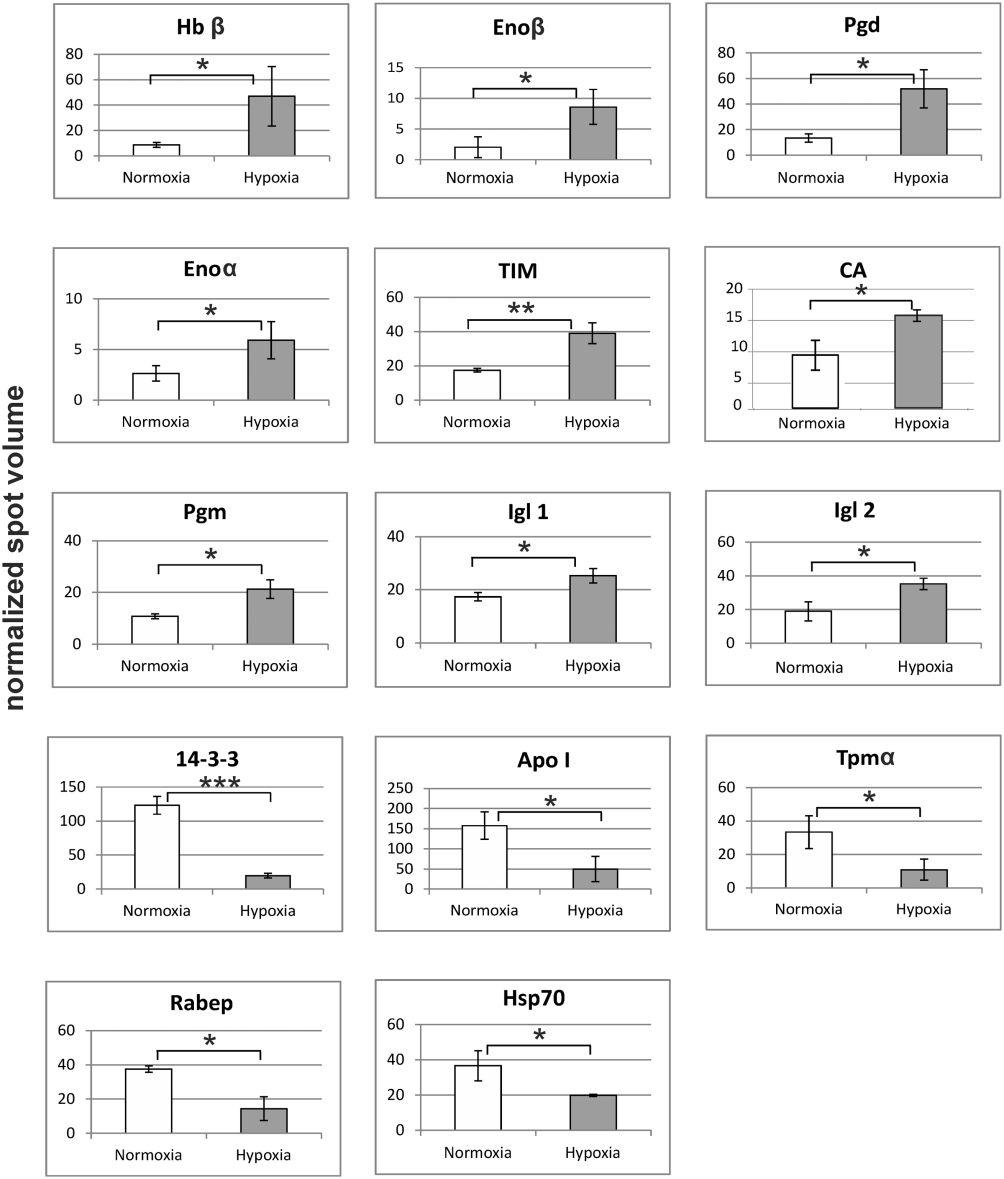
Changes in the protein level in the gills of the ruffe in response to hypoxia. Protein spot volumes were normalized according to GAPDH. Protein expression changes were calculated by the comparison of the mean of hypoxia (n = 3) to normoxia (n = 3) ± s.d., and significance levels were determined by a Student's t-test on the log2 transformed values; * = *p*< 0.1; ** = *p*< 0.01; *** = *p*< 0.001. For detailed information see S6 Table.

After spot detection with the proteomics software Delta 2D, proteins with a more than 1.5-fold up- or down-regulation were identified. In response to hypoxia, 28 of the proteins in the gills of the ruffe (18.8% of the total spots) were found to have significantly different abundance levels and changed at least 1.5-fold (S4 Table). 14 proteins were upregulated, and 14 were downregulated. From the differentially regulated spots, 17 peptide sequences could be obtained by the NanoLC-ESI-ion trap method (see arrows in Fig 3A; S2 Appendix). These peptide sequences were then used to identify the corresponding gene sequence in the transcriptome of the ruffe, in the UniProtKB and the NCBI database, which was successful for 14 protein spots (S6 Table; Fig 4). The strongest response to hypoxia was found for hemoglobin β (Hbβ), which changed 5.3-fold. Five proteins that are involved in the energy metabolism were found upregulated, including four enzymes of the glycolysis, *i*.*e*. enolase α and β (Enoα + β; 4.2-fold), triosephosphate isomerase (TIM; 2.2-fold), and phosphoglycerate mutase (PGM; 1.9-fold). The levels of 6-phosphogluconat-dehydrogenase (Pdg), which is involved in the pentose phosphate pathway, increased 2.9-fold. Further, we found the levels of carbonic anhydrase (CA; 1.8-fold) and two domains from immunoglobulin light chain (Igl 1 and 2; 1.46- and 1.86-fold) increased. A three to fivefold downregulation in response to hypoxia was observed for two proteins that play a role in cell signalling: rab GTPase-binding effector protein 2-like (Rabep) and 14-3-3-protein. The other downregulated proteins include apolipoprotein (Apo I; 2.2-fold) and the molecular chaperone heat shock protein 70 (Hsp70), which both decreased about threefold.

**Fig 5:**
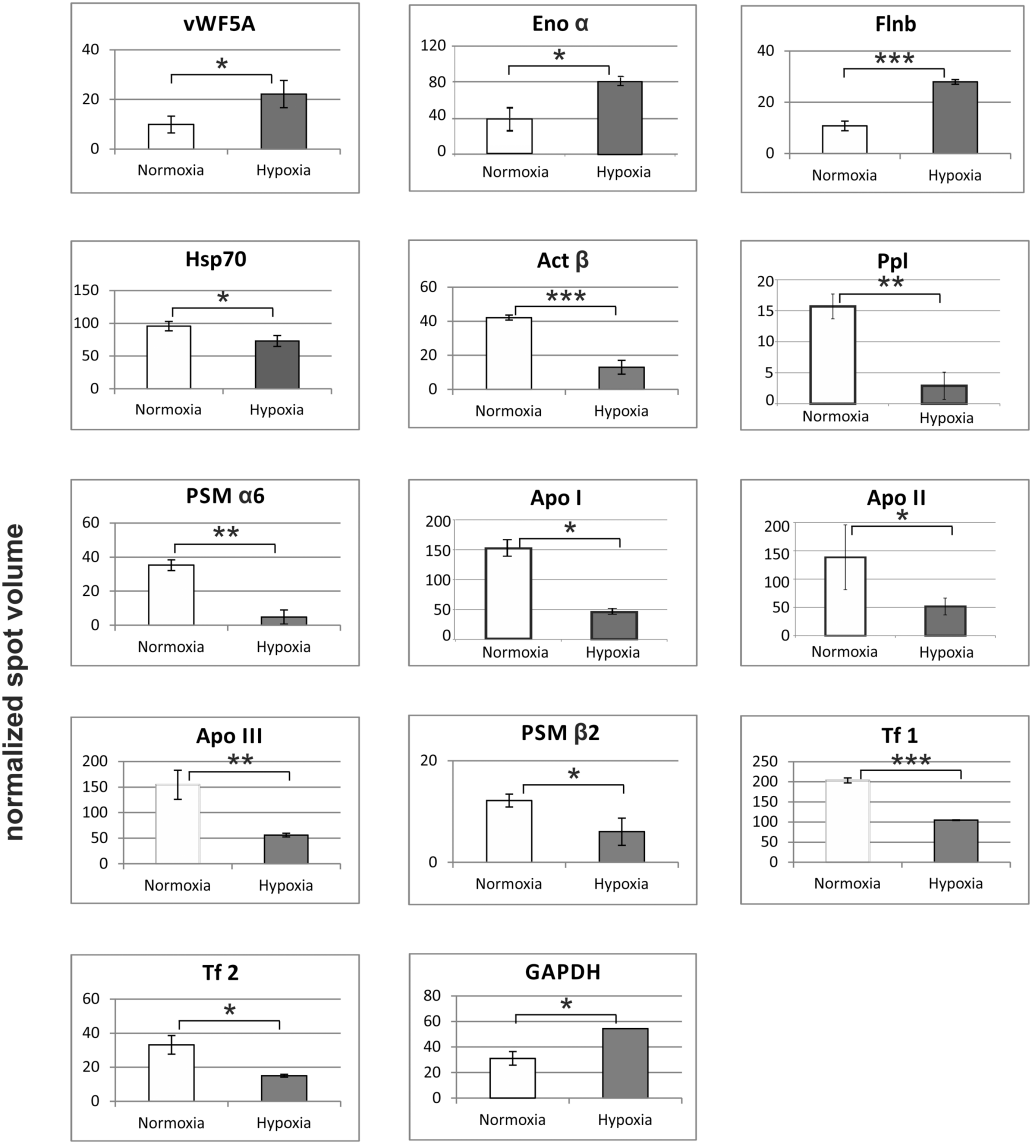
Changes in the protein level in the gills of the European flounder in response to hypoxia. Protein spot volumes were normalized according to GAPDH. Protein expression changes were calculated by the comparison of the mean of hypoxia (n = 3) to normoxia (n = 3) ± s.d., and significance levels were determined by a Student's t-test on the log2 transformed values; * = *p*< 0.1; ** = *p*< 0.01; *** = *p*< 0.001. For detailed information see S7 Table.

Hypoxia-induced significant changes in the abundance levels of 27 spots (22.3% of total spots) in the gills of the flounder, of which six were upregulated, and 21 downregulated (S5 Table). 14 protein spots could be identified by mass spectrometry (S7 Table; S3 Appendix). Three of the identified proteins were found upregulated (Fig 5): filamin B-like (Flnb; 2.59-fold), von Willebrand factor (vWR5a; 2.24-fold) and enolase α (Enoα; 2.02-fold). The strongest downregulation (about sevenfold) was found for the proteasome subunit α6 (PSM α6); another proteasome subunit (PSM β2) was downregulated twofold. Two proteins related to the cytoskeleton were downregulated three- to fivefold: actin β (β-Act) and periplakin (Ppl). Hypoxia exposure reduced the levels of apolipoproteins I, II and III (Apo I, II and III) about threefold, and the levels of two transferrins (Tf) about twofold. Hypoxia further reduced the level of heat shock protein 70 (Hsp70); however, proteomics data did not allow discriminating between different HSP70 isoforms.

### Effects of hypoxia on mRNA levels in ruffe and flounder gills

**Fig 6:**
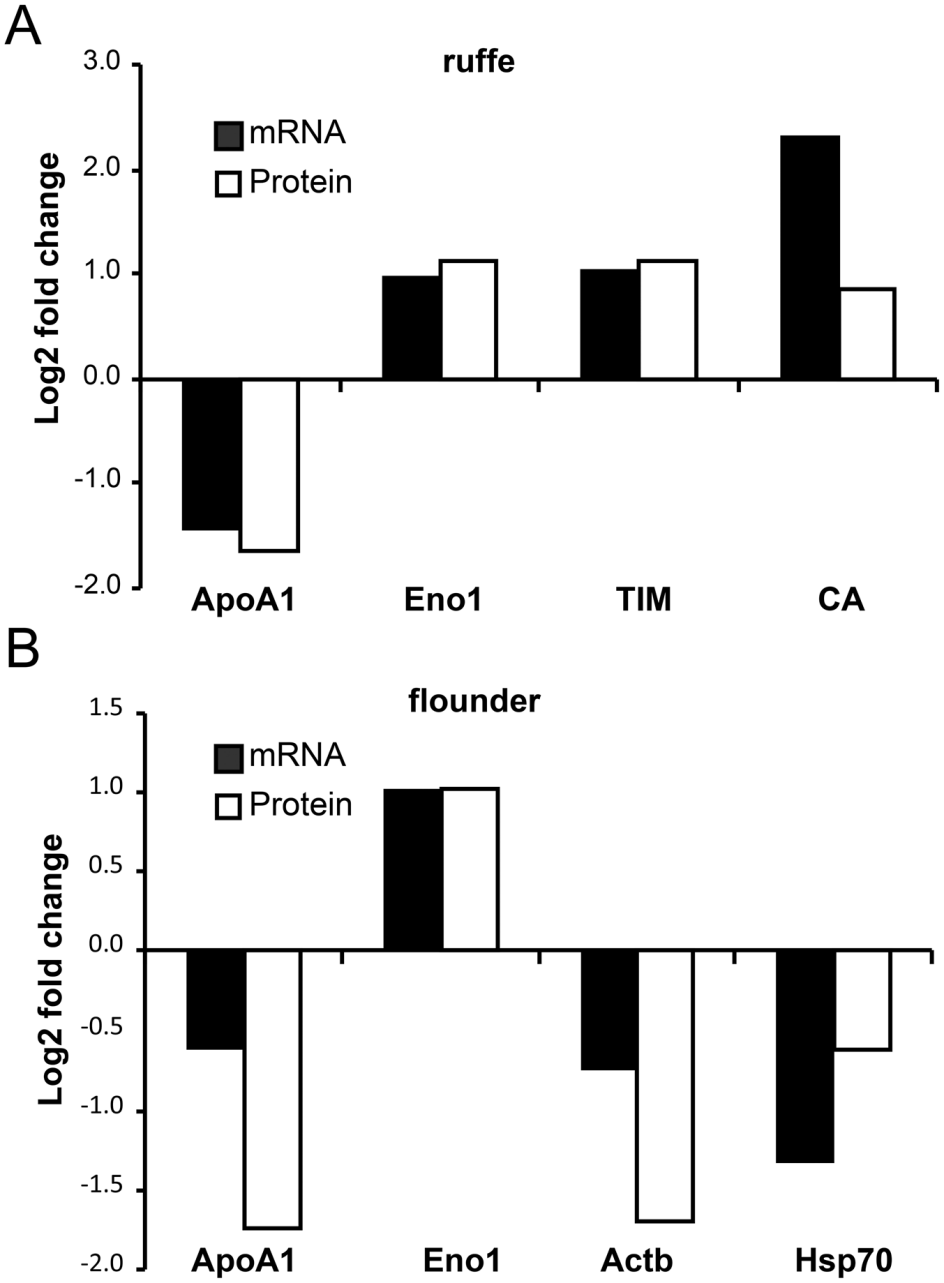
Comparison of the changes in protein and mRNA levels in the gills of ruffe (A) and flounder (B). Changes in protein levels were calculated as stated above (see Figs 4 and 5). mRNA copy numbers were measured by qRT-PCR (n = 3). The data are presented as log2 fold changes. The raw qRT-PCR data are provided in S8 table, the standard deviations for the proteins are given in S6 and S7 tables. A scatter plot that compares the changes of protein and mRNA levels is provided as S2 Fig.

We further investigated the molecular response in the gills to hypoxia using qRT-PCR. We selected four genes from each, ruffe and European flounder, on the basis of the proteome data (Figs 4 and 5; S6 and S7 tables; the raw qRT-PCR data and amplification efficiencies are given in S8 table). The genes Eno α and ApoA1 were selected because they changed in both species. There are multiple contigs that may correspond to the HSP70 found in proteomics. We used for the design of the primers the flounder ortholog of *D*. *rerio* Hsp70 protein 8 (EMBL/GenBank acc. no. AY422994), which corresponds to a constitutively HSP70 in mammals and that has been previously used in hypoxia studies with flounder [25]. For each gene, the mRNA levels of samples from normoxic and hypoxic gills were compared and the fold-changes calculated. These values were compared with those estimated from the proteomic data (Figs 4 and 5). For most of the studied genes, the observed changes in the mRNA and protein levels match, with concordant direction of regulation in all of them (Fig 6). In the ruffe, the numbers were essentially identical, with the exception of CA, for which about fivefold more mRNA was observed in the hypoxic gills, while the protein levels differ only by a factor of two. In the European flounder, the main difference was found in the levels of Enoα, which showed an about twofold increase in mRNA, but an 8.75-fold increase in protein levels.

## Discussion

### The transcriptomes of ruffe and flounder enhanced protein detection by mass spectrometry

Transcriptomes were generated from mixed tissues of the ruffe and the European flounder. These two fishes are key species in the Elbe estuary, but only a few sequences have been reported so far. Illumina transcriptome sequencing is an easily feasible and rather inexpensive method that allows the efficient discovery of genes (Table 2). The mixed tissue approach (instead of specifically sequencing the gill transcriptomes) was chosen to cover a broad range of genes. Nevertheless, we found only about 60 to 70% overlap in the sequenced genes between both species, as estimated from the comparisons with the human and zebrafish protein databases (Fig 1) and from reciprocal blastn searches (S1 Fig; S2 table). This discrepancy probably reflects the species-specific expression of some genes. Despite that, GO analyses showed that the distribution of the identified transcripts into functional categories was largely similar (Fig 2).

In this study, species-specific transcriptomes were employed in addition to the UniProtKB and NCBI nr databases for the identification of proteins by mass spectrometry. This enhanced the detection levels of this method, fascilitating the identifiaction of four additional proteins in the ruffe (S6 Table) and one protein in the flounder (S7 table).

### Evaluating the hypoxia response of ruffe and flounder gill proteomes

The characterisation of the molecular basis of stress tolerance against naturally shifting environmental conditions has great physiological and pathophysiological significance. Several proteome studies on the response to natural stressors have suggested that a conserved group of stress-induced proteins exists over a wide range of taxa [45, 46]. These proteins are mainly involved in protection and repair mechanisms of macromolecules such as proteins, nucleic acids and lipids [45]. In addition, previous studies have suggested that adaptation processes to hypoxia are often associated with a reduction of energy-consuming processes and a switch from aerobic to glycolytic energy production in hypoxia-tolerant fish species [18, 47].

To better understand the functional changes in the gills of ruffe and flounder in response to severe hypoxia, we employed a proteome approach, which was supported by transcriptome data, and qRT-PCR analysis. The results provide new insights into the adaptation processes of these estuarine fish species to hypoxia, which is a recurring phenomenon in their natural habitats [7–10]. The protein patterns in the gills provide information on the mechanisms of hypoxia-adaptation in these species, and the differences may hint to species-specificity of the hypoxia response, which may be explained by the different lifestyles of the flounder and the ruffe.

### Hypoxia induces the anaerobic energy metabolism in the gills

It is well established that in many vertebrates, including fish, hypoxia induces a shift from the oxidative metabolism and the preference of lipids to an anaerobic metabolism and the preference of carbohydrates [48]. Our data suggest that in the gills of both the ruffe and the flounder, enzymes that are involved in the energy metabolism represent the majority of the proteins that changed in response to hypoxia. At the first glance, this seems surprising because the gills are expected to be the best-oxygenated organs. On the one hand, it must be considered that the identified proteins only represent a small fraction of genes that changed under hypoxia. On the other hand, however, the extraction of O_2_ from the water is a very energy-demanding process, and gills are also in charge of osmoregulation [49, 50]. Therefore, they consume a large fraction of the energy, and a preferentially anaerobic energy metabolism may help to sustain gill functions during periods of reduced O_2_ availability.

The increase of the levels of glycolytic enzymes (Enoα+β, TIM, Pgm) agrees with previous observations that under hypoxia most teleosts switch to glycolysis [24, 27, 51–53]. We also found in the gills of the ruffe an increase of Pgd, an enzyme from the pentose phosphate pathway, which helps to maintain the cellular NADPH level. Moreover, we observed decreased levels of apolipoproteins in the gills of both species, three isoforms in the flounder (Apo I, Apo II and Apo III) and one in the ruffe (Apo I). The main function of apolipoproteins is the transport of lipids. One possible explanation is that under hypoxic conditions, less oxidative metabolism occurs [20] and lipids are required for energy production by β-oxidation. Therefore, a reduction of lipid transport to the gills may be expected. It should also be noted that at least some of the apolipoproteins may derive from the blood although the gills have been rinsed before use.

One physiological hallmark of the hypoxia response in fish is an increase in hematocrit, which is mostly due to an increase in red blood cell formation [19, 54]. In the ruffe, Hbβ showed the strongest effect at hypoxia and increased 6.75-fold. This may be either explained by an increased blood flow or by the induction of a specific β-chain that alters the affinity of hemoglobin to O_2_. By contrast, in the flounder, we found decreased levels of transferrin (Tf), which transports iron and is thus involved in the synthesis of hemoglobin. At the first glance, this is surprising because Tf is considered as a hypoxia-inducible gene at least in the mammalian system [55]. A reduced level of Tf is an indicator for a general reduction in hemoglobin synthesis. It should be noted that our data on hemoglobin and Tf regulation derive from different species. It may be possible, that hemoglobin levels are differentially regulated under hypoxia in different fish species [16, 17, 56]. Additional studies are required to clarify this issue.

In the ruffe gills, two of the proteins that change in response to hypoxia either belong to the G-protein family (Rabep) or are involved in their regulation (14-3-3). G-proteins are essential parts of the signal transduction, where they are the mediators between membrane receptors and the second messenger system [57]. When proteins of the Ras superfamily are activated, they induce cell growth, differentiation and survival processes. Hence, our data may be interpreted as an indicator that hypoxia reduces cell proliferation, which agrees with previous studies employing fish [18, 20].

It is well known that hypoxia and other environmental stresses lead to a conserved cellular response in fish, which is triggered by damage to macromolecules [58–60]. The major aspects of the molecular stress response are the maintenance of cellular homeostasis and macromolecular integrity, which is accomplished by molecular chaperones and DNA repair proteins but also the removal of macromolecular debris by the proteasome [45]. Notably, we found in both species a reduction of Hsp70 levels in the gills. Hsp70 regulates the correct folding, prevents the aggregation of damaged proteins and also cooperates with the proteasome in protein degradation. Decreased mRNA levels of proteasome-related genes have already been shown during the hypoxia adaptation in zebrafish [20]. Notably, in a previous study we found divergent regulation patterns of Hsp70 in both species, which showed a strong dependence on the life history of the specimens (laboratory vs. field data) and the degree of hypoxia [25].

### Species-specificity of hypoxia response

One of the major factors that determine the hypoxia tolerance and hypoxia response of a species is its lifestyle. The flounder is a demersal fish species that experiences hypoxic periods in its natural habitat [58]. It has already been demonstrated that the flounder shows a remarkable tolerance towards hypoxia [59, 61], accompanied by pronounced changes in gene expression patterns in response to hypoxia, particularly in the gills [25]. The ruffe is also defined as a hypoxia-tolerant species [32] but showed a somewhat weaker response on the level of selected hypoxia-related genes [25]. In our proteomics approach, we found that the number of proteins in the gills that significantly changed in response to hypoxia was similar in the ruffe and the flounder (28 vs. 27). Thus at least on the level of the proteins with the strongest change in abundance, and by their numbers, the inter-species differences are less pronounced than on the level of specifically selected genes [25].

We must further consider that, in both species, some of the proteins that showed increased abundance might reflect an increase in blood flow in the gills in response to hypoxia. This may be true e.g. for hemoglobin, but possibly also the carbonic anhydrase. Although—compared to the qRT-PCR approach with specifically selected genes—a proteome approach has clear advantages in being unbiased by *a priori* assumptions, such factors need to be considered.

## Supporting Information

S1 Appendix 2D gels of the gill proteome of the ruffe and the European flounder. For each species, three gels that derive from hypoxia treatment and three gels from the normoxia controls are supplied.
(ZIP)

S2 Appendix Mass spectrometry data from the protein spots of the ruffe gills. The peak lists are in the mzML format. For the numbers of the protein spots, see Fig 3A.
(ZIP)

S3 Appendix Mass spectrometry data from the protein spots of the flounder gills. The peak lists are in the mzML format. For the numbers of the protein spots, see Fig 3B.
(ZIP)

S1 Fig Comparison of ruffe and European flounder transcriptomes by reciprocal blast search. The Venn diagram shows the number of sequences identified in both transcriptomes by a reciprocal blastn search with an e-value cut-off of 1e-10. The overlap represents the number of reciprocal contigs, i.e., the contig pair was found in the blastn searches regardless of being query or database entry.
(PDF)

S2 Fig Scatter plot comparison of the changes in protein and mRNA levels in the gills of ruffe (A) and flounder (B). Changes in protein levels were calculated as described in the text (see Figs 4 and 5), the mRNA levels were measured by qRT-PCR (Fig 6).
(PDF)

S1 Table Primer sequences and amplicon sizes of the genes used for qRT-PCR experiments. (DOCX)

S2 Table Comparison of ruffe and European flounder transcriptomes. The table shows the results of the reciprocal blastn analyses with an e-value cut-off of 1e-10.
(DOC)

S3 Table PANTHER analysis of ruffe and flounder transcriptomes. The GO terms were derived from the blastx annotated contigs of ruffe and flounder against the human database.
(DOC)

S4 Table Proteins that changed under hypoxia in the ruffe gills (cf. Fig 3A). (DOC)

S5 Table Proteins that changed under hypoxia in the flounder gills (cf. Fig 3B). (DOC)

S6 Table Protein identifications and abundance ratios in response to hypoxia in the gills of the ruffe. (DOC)

S7 Table Protein identifications and abundance ratios in response to hypoxia in the gills of the flounder. (DOC)

S8 Table Raw qRT-PCR results and amplification efficiencies. (XLS)
