# Supplementary figures and images for: Evaluating the Hypoxia Response of Ruffe and Flounder Gills by a Combined Proteome and Transcriptome Approach
Source: PLoS One. 2015 Aug 14;10(8):e0135911. doi: 10.1371/journal.pone.0135911 (PMC4537130; doi:10.1371/journal.pone.0135911)

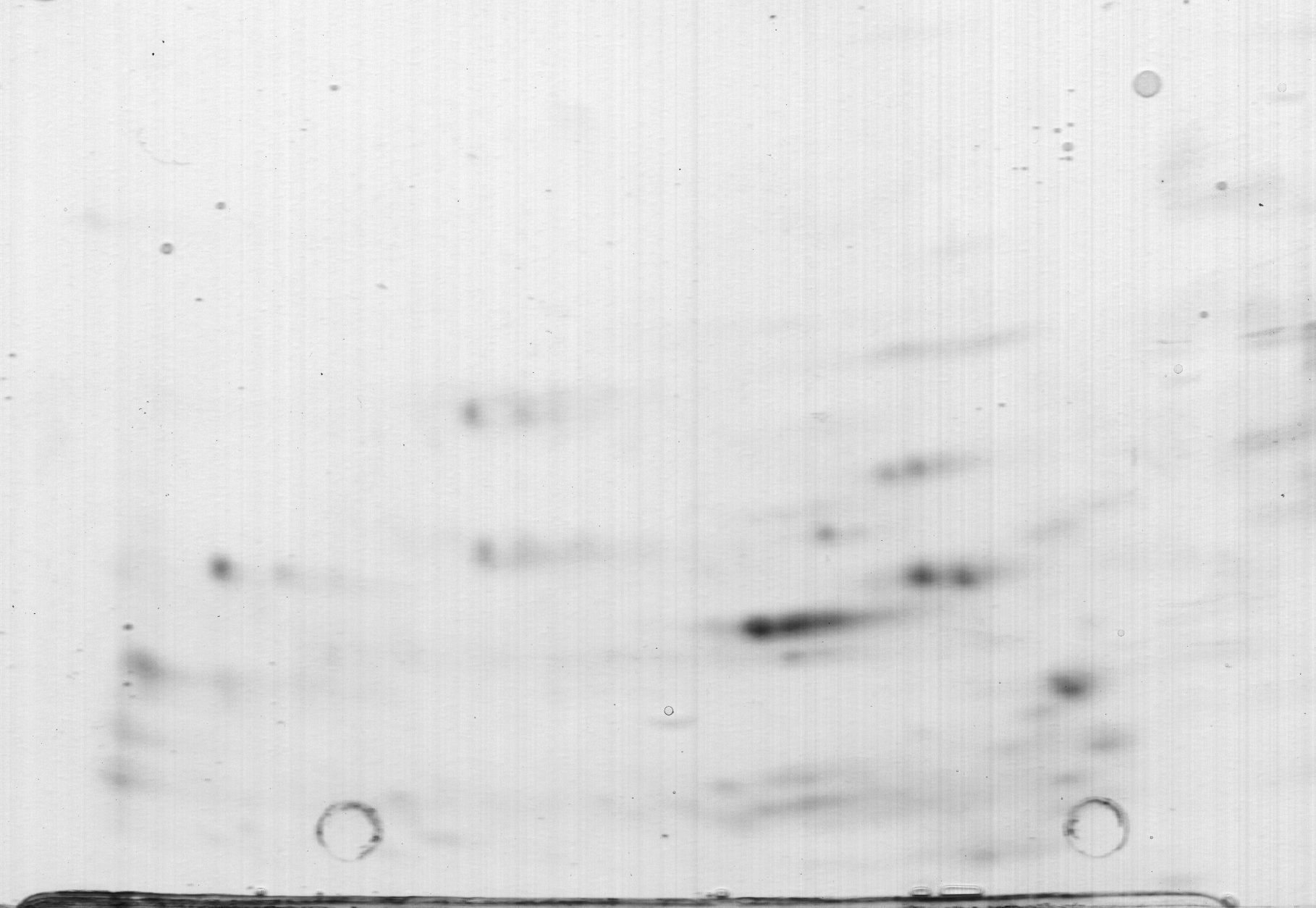

Supplement: S1 Appendix — For each species, three gels that derive from hypoxia treatment and three gels from the normoxia controls are supplied. (ZIP) [file pone.0135911.s001.zip › 2D Gels/Flounder Hypoxia 1.tif]

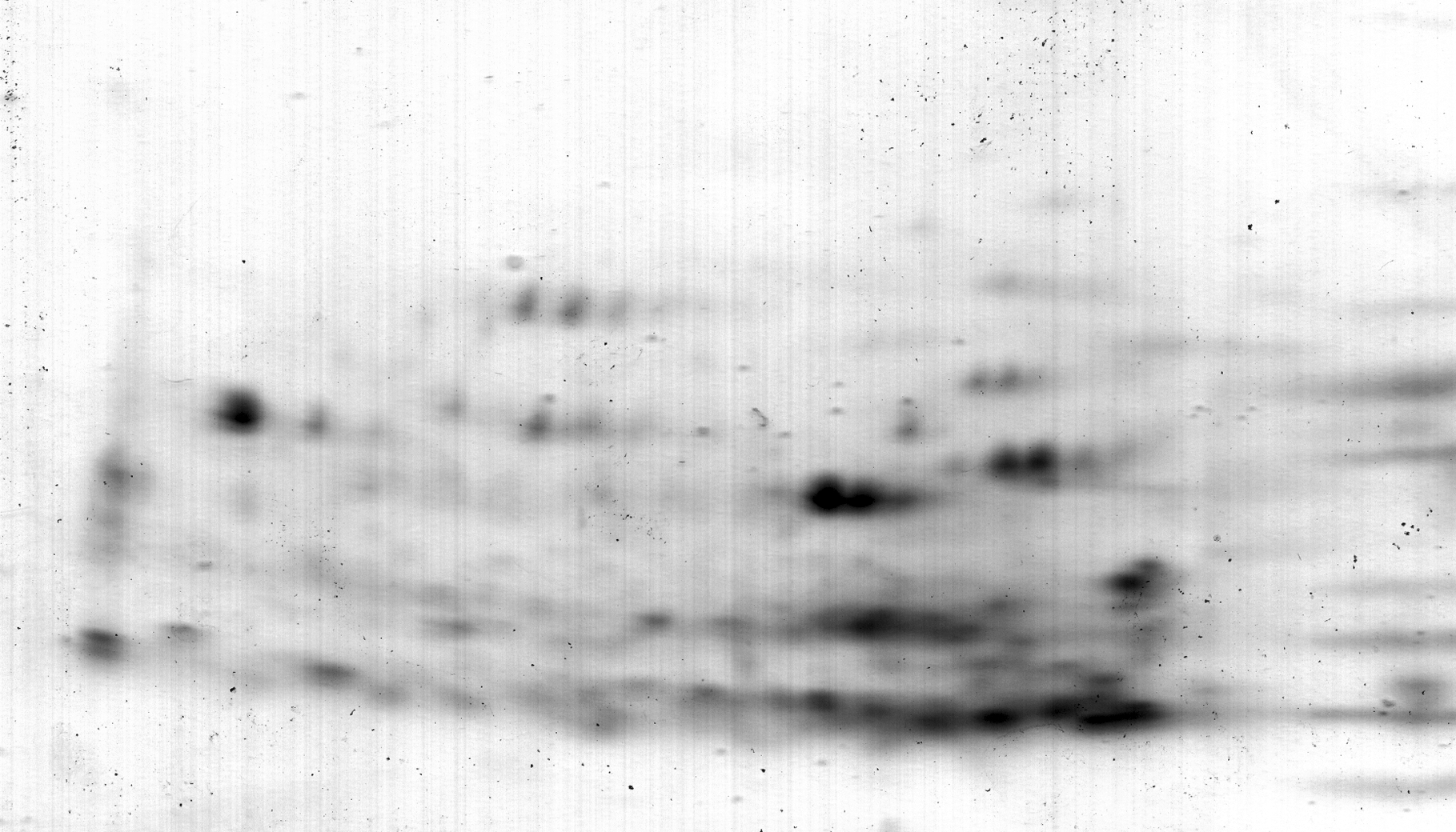

Supplement: S1 Appendix — For each species, three gels that derive from hypoxia treatment and three gels from the normoxia controls are supplied. (ZIP) [file pone.0135911.s001.zip › 2D Gels/Flounder Hypoxia 2.tif]

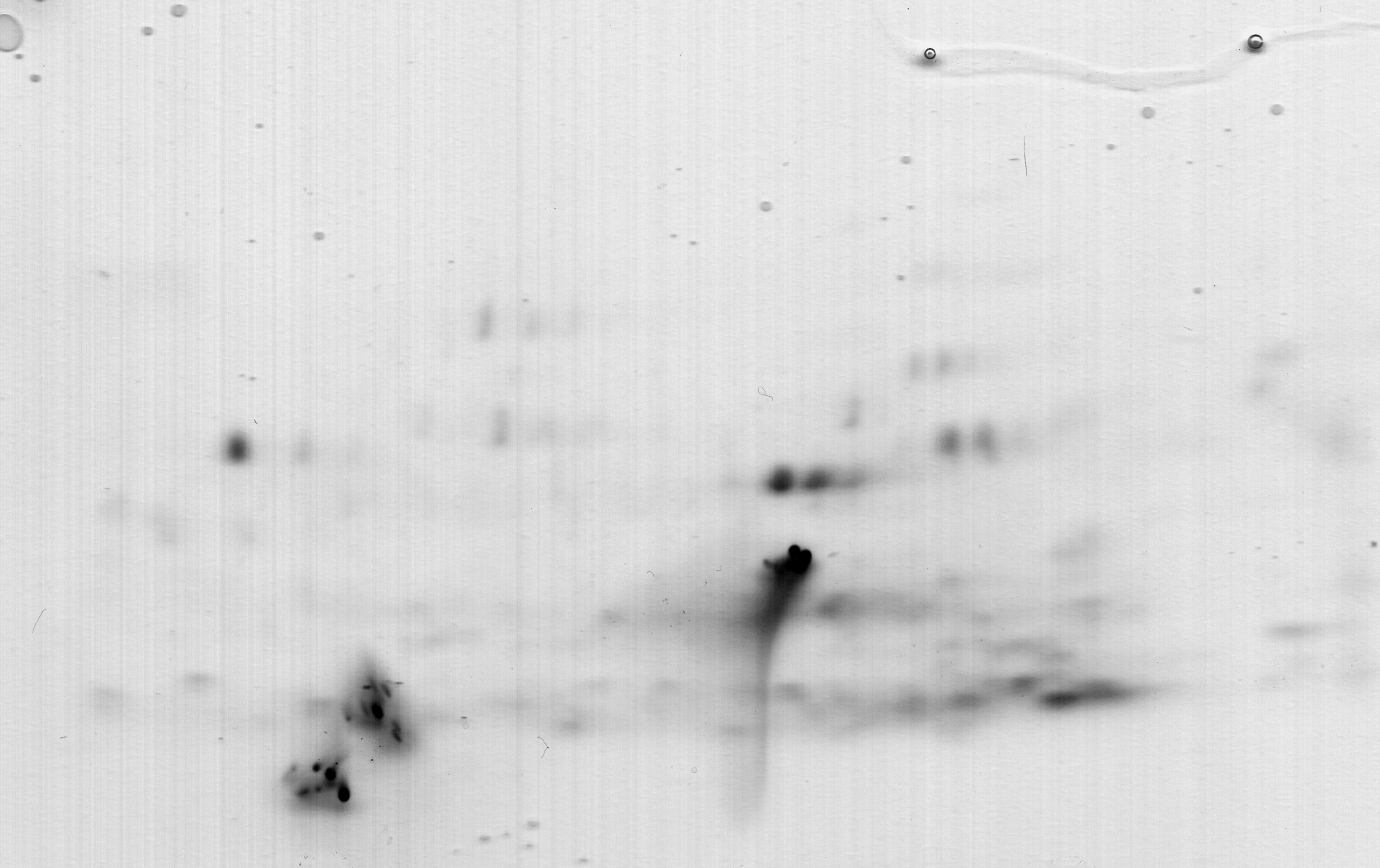

Supplement: S1 Appendix — For each species, three gels that derive from hypoxia treatment and three gels from the normoxia controls are supplied. (ZIP) [file pone.0135911.s001.zip › 2D Gels/Flounder Hypoxia 3.tif]

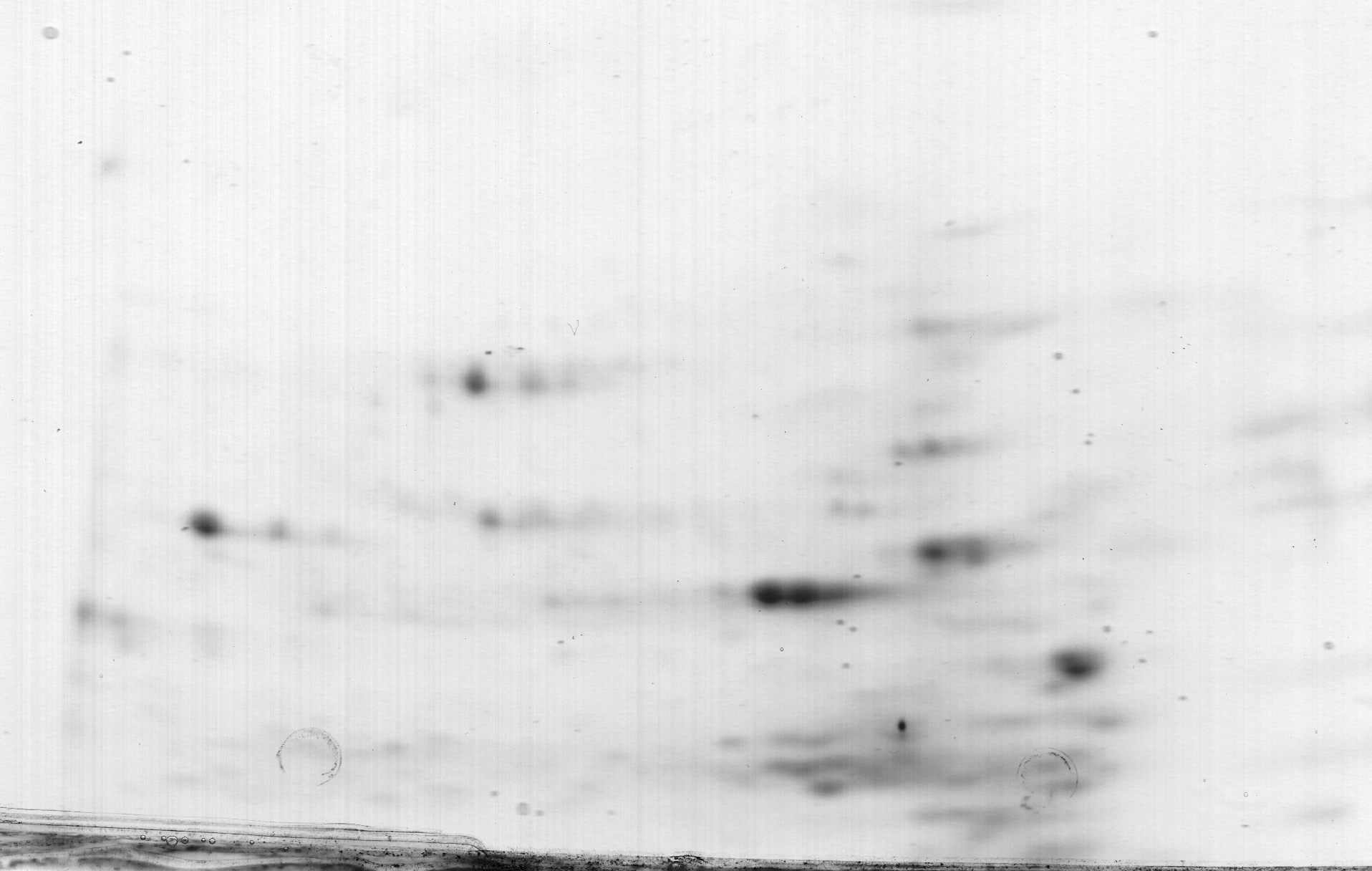

Supplement: S1 Appendix — For each species, three gels that derive from hypoxia treatment and three gels from the normoxia controls are supplied. (ZIP) [file pone.0135911.s001.zip › 2D Gels/Flounder Normoxia 1.tif]

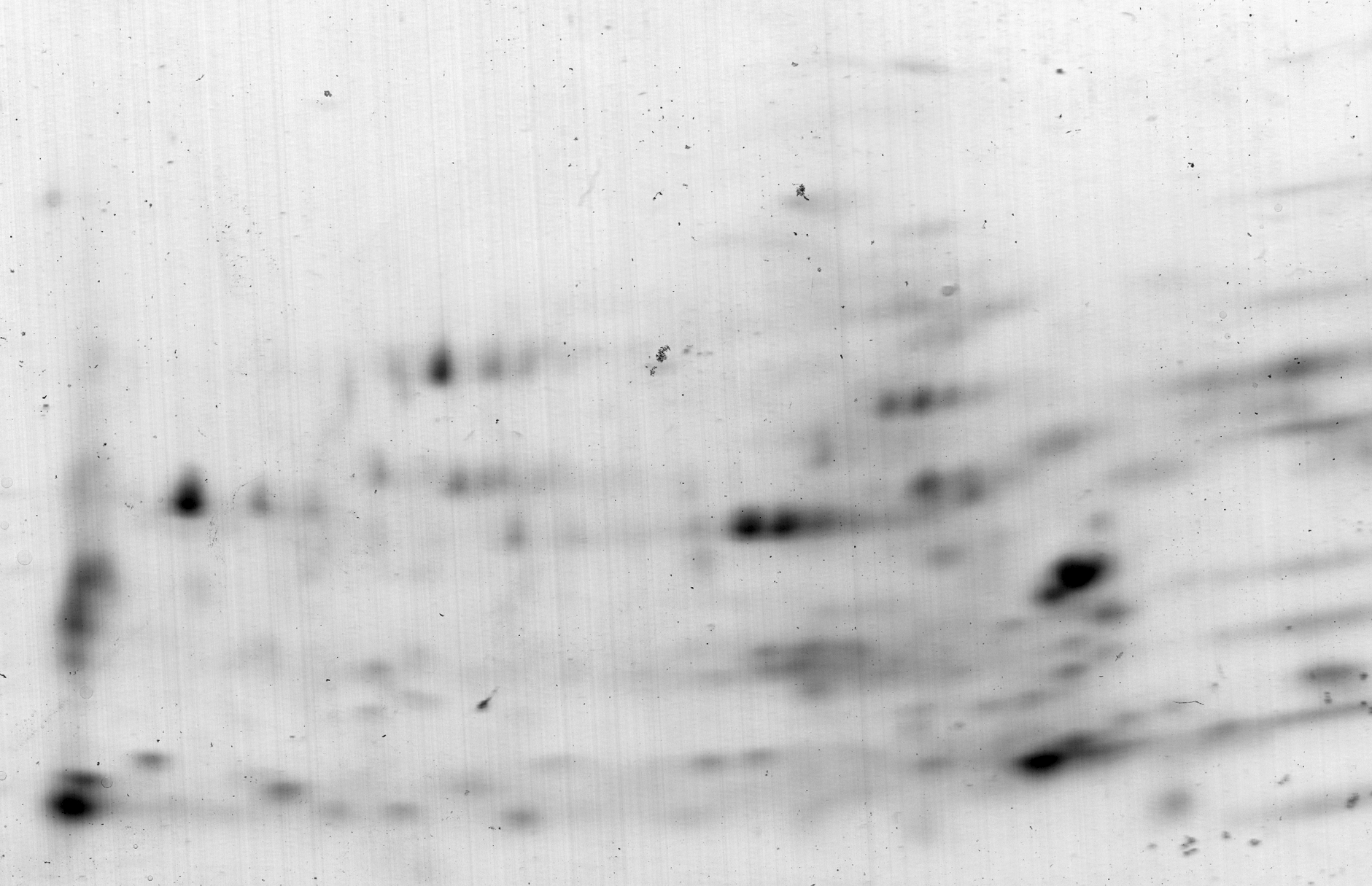

Supplement: S1 Appendix — For each species, three gels that derive from hypoxia treatment and three gels from the normoxia controls are supplied. (ZIP) [file pone.0135911.s001.zip › 2D Gels/Flounder Normoxia 2.tif]

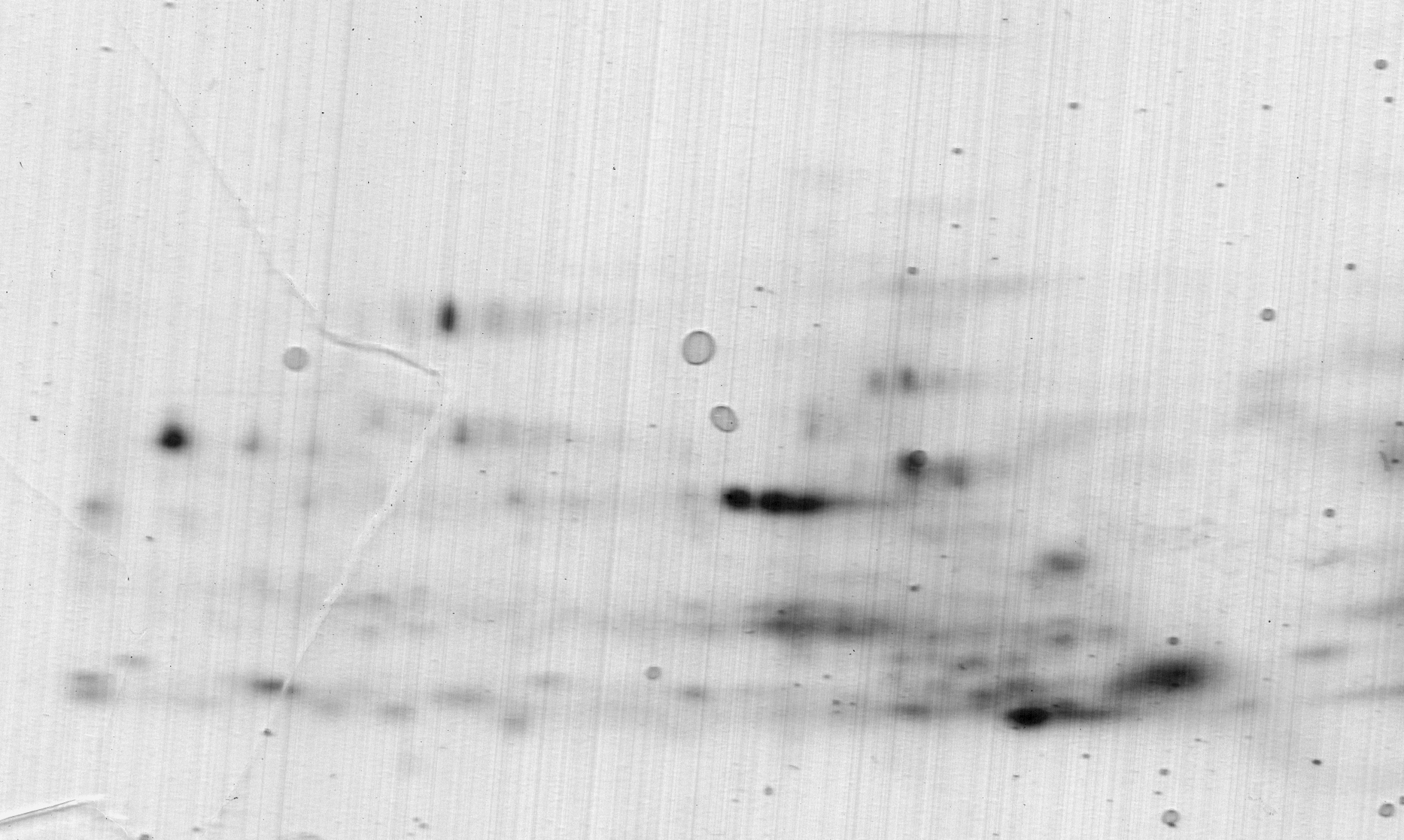

Supplement: S1 Appendix — For each species, three gels that derive from hypoxia treatment and three gels from the normoxia controls are supplied. (ZIP) [file pone.0135911.s001.zip › 2D Gels/Flounder Normoxia 3.tif]

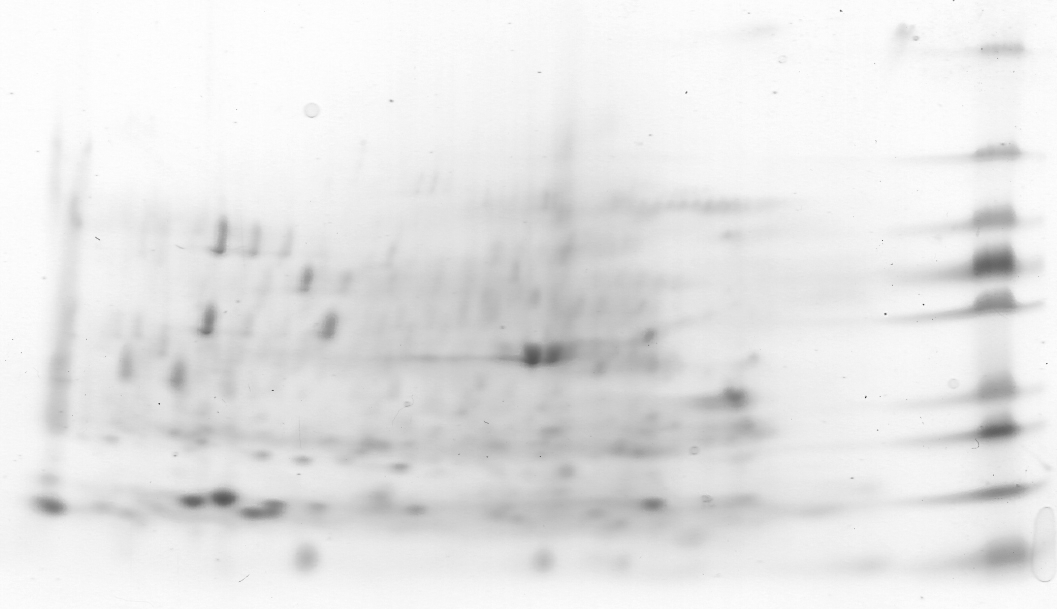

Supplement: S1 Appendix — For each species, three gels that derive from hypoxia treatment and three gels from the normoxia controls are supplied. (ZIP) [file pone.0135911.s001.zip › 2D Gels/Ruffe Hypoxia 1.tif]

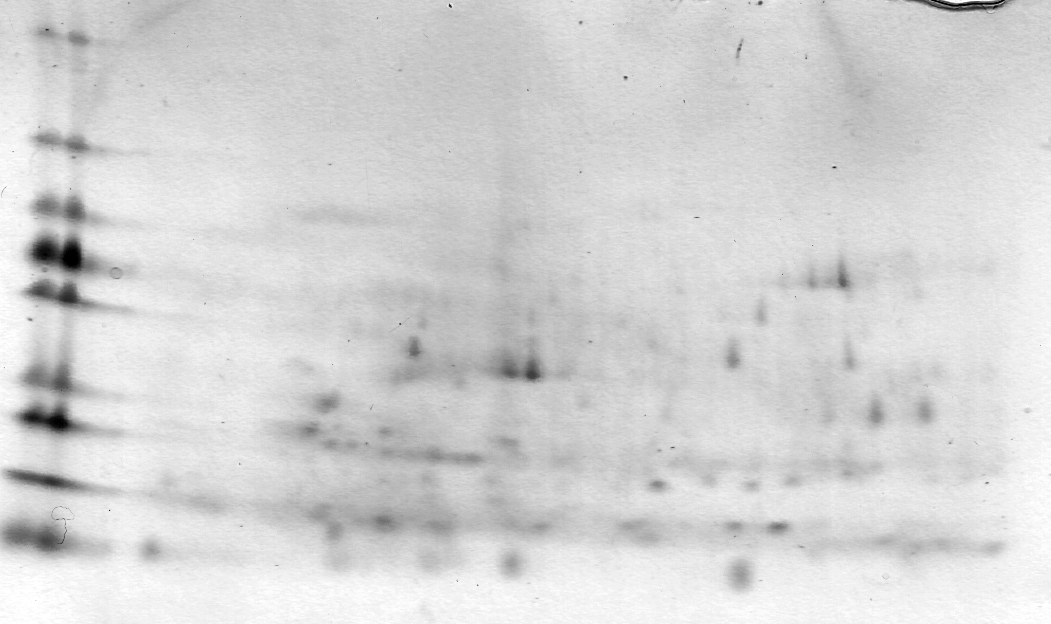

Supplement: S1 Appendix — For each species, three gels that derive from hypoxia treatment and three gels from the normoxia controls are supplied. (ZIP) [file pone.0135911.s001.zip › 2D Gels/Ruffe Hypoxia 2.tif]

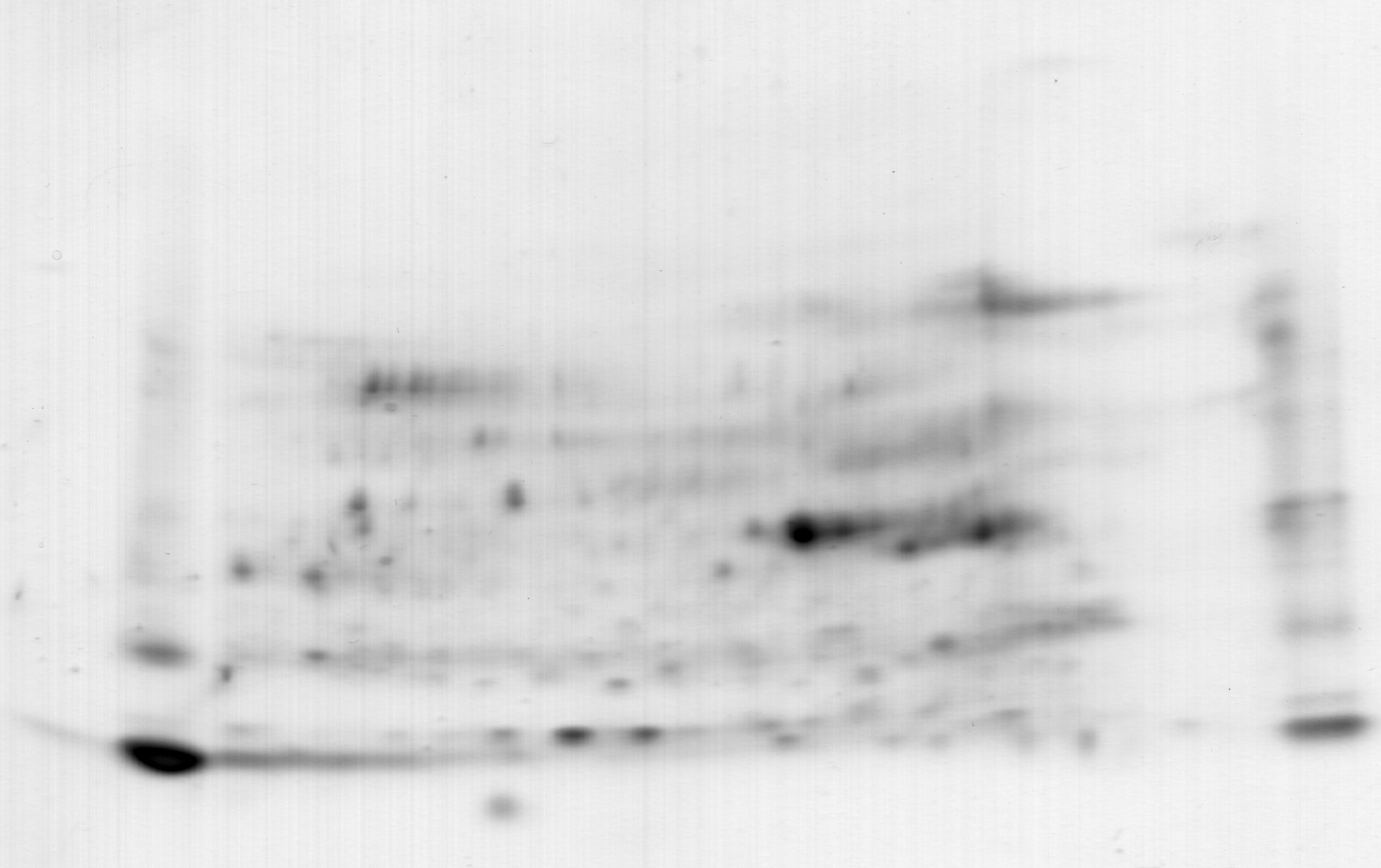

Supplement: S1 Appendix — For each species, three gels that derive from hypoxia treatment and three gels from the normoxia controls are supplied. (ZIP) [file pone.0135911.s001.zip › 2D Gels/Ruffe Hypoxia 3.tif]

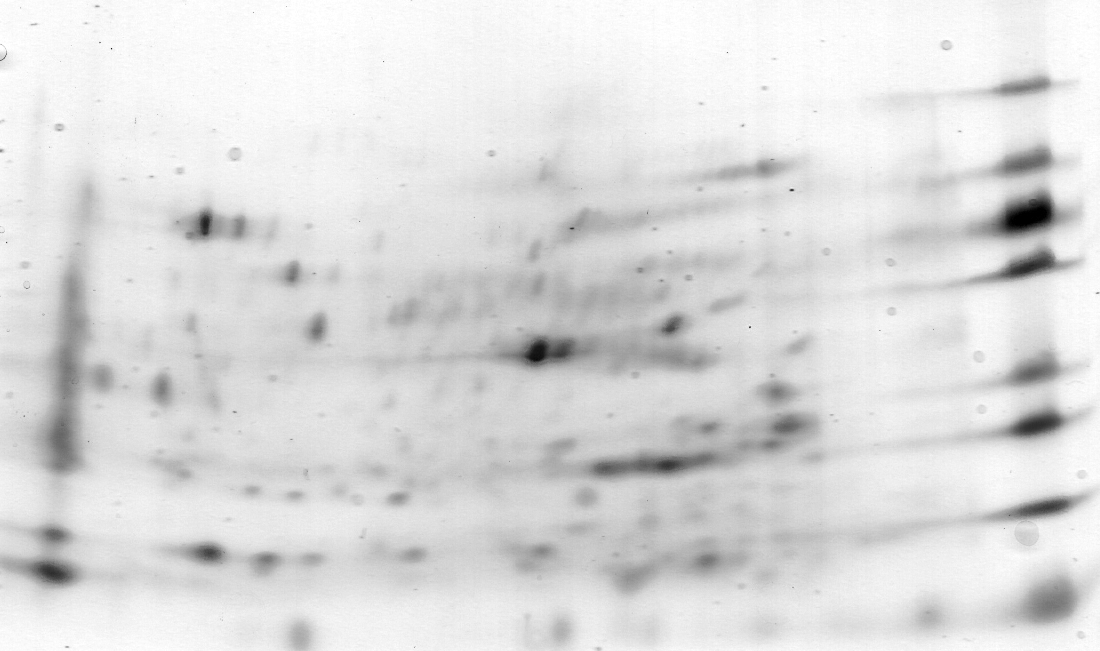

Supplement: S1 Appendix — For each species, three gels that derive from hypoxia treatment and three gels from the normoxia controls are supplied. (ZIP) [file pone.0135911.s001.zip › 2D Gels/Ruffe Normoxia 1.tif]

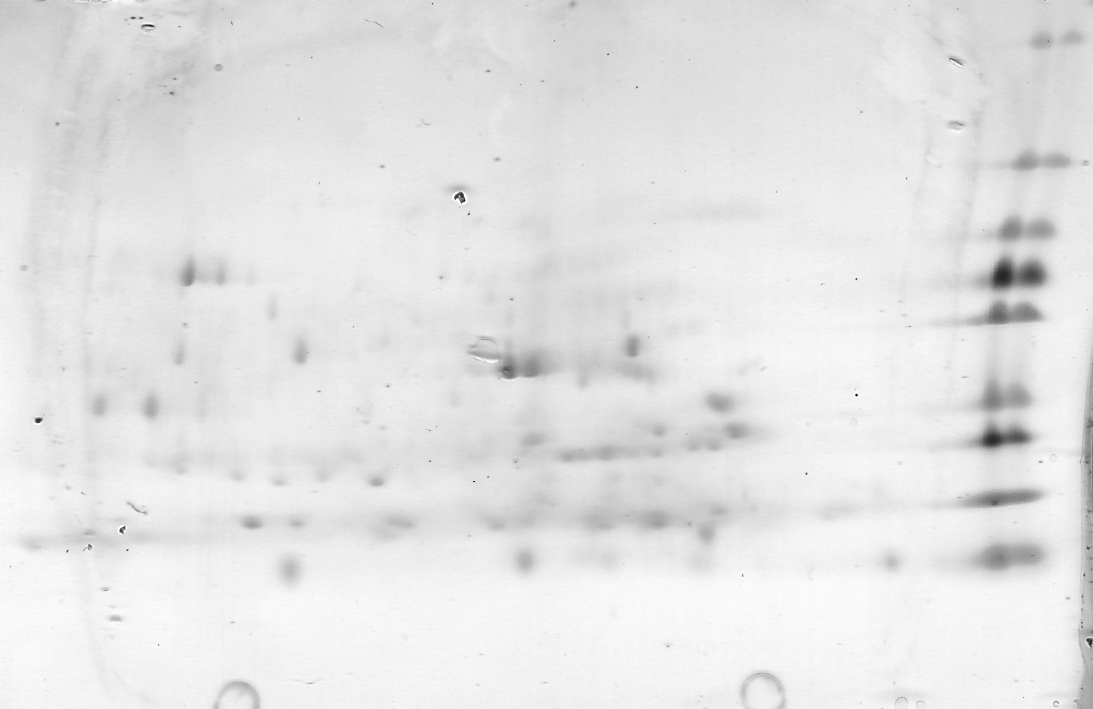

Supplement: S1 Appendix — For each species, three gels that derive from hypoxia treatment and three gels from the normoxia controls are supplied. (ZIP) [file pone.0135911.s001.zip › 2D Gels/Ruffe Normoxia 2.tif]

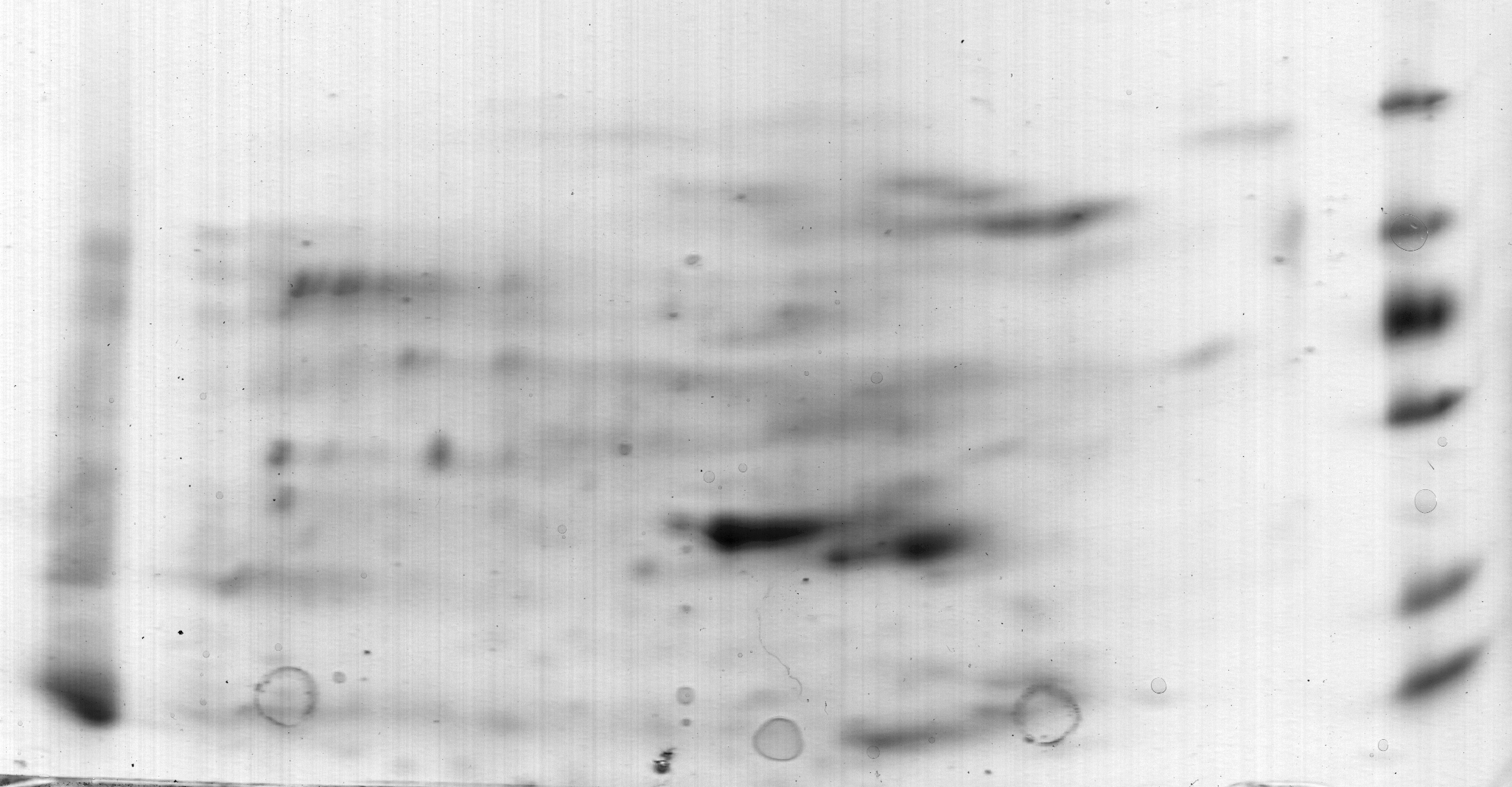

Supplement: S1 Appendix — For each species, three gels that derive from hypoxia treatment and three gels from the normoxia controls are supplied. (ZIP) [file pone.0135911.s001.zip › 2D Gels/Ruffe Normoxia 3.tif]
