# Supplementary material for: Evaluating the Hypoxia Response of Ruffe and Flounder Gills by a Combined Proteome and Transcriptome Approach
Source: PLoS One. 2015 Aug 14;10(8):e0135911. doi: 10.1371/journal.pone.0135911 (PMC4537130; doi:10.1371/journal.pone.0135911)
Supplement: S1 Fig — The Venn diagram shows the number of sequences identified in both transcriptomes by a reciprocal blastn search with an e-value cut-off of 1e-10. The overlap represents the number of reciprocal contigs, i.e., the contig pair was found in the blastn searches regardless of being query or database entry. (PDF) [file pone.0135911.s004.pdf]

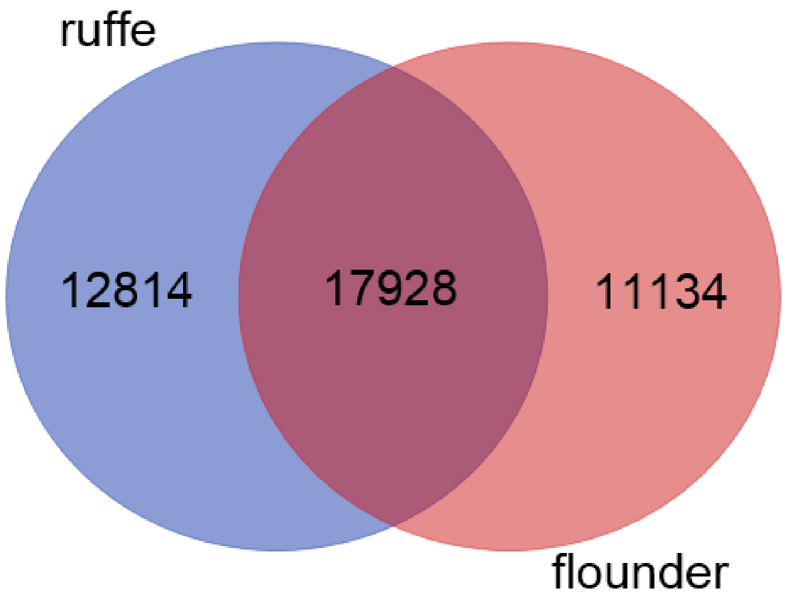

S1 Figure. Comparison of ruffe and European flounder transcriptomes by reciprocal blast search. The Venn diagram shows the number of sequences identified in both transcriptomes by a reciprocal blastn search with an e-value cut-off of 1e-10. The overlap represents the number of reciprocal contigs, i.e., the contig pair was found in the blastn searches regardless of being query or database entry.
