# Supplementary material for: Evaluating the Hypoxia Response of Ruffe and Flounder Gills by a Combined Proteome and Transcriptome Approach
Source: PLoS One. 2015 Aug 14;10(8):e0135911. doi: 10.1371/journal.pone.0135911 (PMC4537130; doi:10.1371/journal.pone.0135911)
Supplement: S2 Fig — Changes in protein levels were calculated as described in the text (see Figs 4 and 5), the mRNA levels were measured by qRT-PCR (Fig 6). (PDF) [file pone.0135911.s005.pdf]

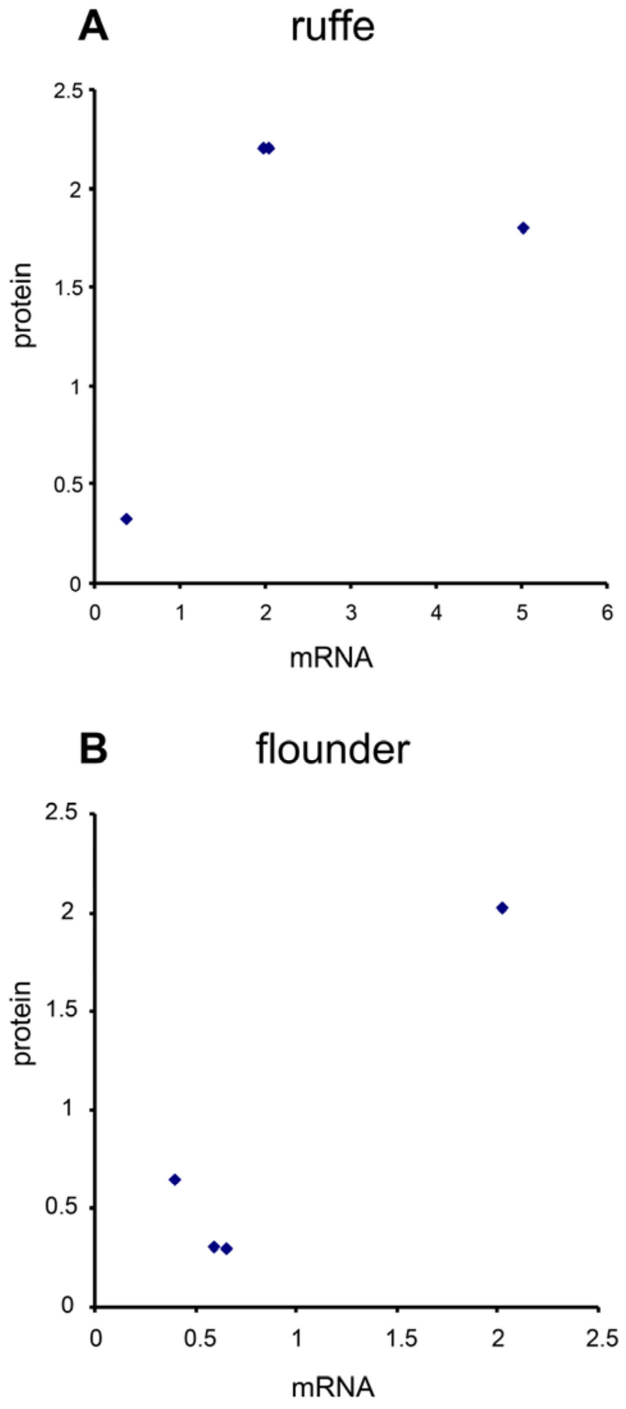

S2 Figure. Scatter plot comparison of the changes in protein and mRNA levels in the gills of ruffe and flounder. Changes in protein levels were calculated as described in the text (see Figs. 4 and 5), the mRNA levels were measured by qRT-PCR (Fig. 6).
