# Supplementary material for: Evaluating the Hypoxia Response of Ruffe and Flounder Gills by a Combined Proteome and Transcriptome Approach
Source: PLoS One. 2015 Aug 14;10(8):e0135911. doi: 10.1371/journal.pone.0135911 (PMC4537130; doi:10.1371/journal.pone.0135911)
Supplement: S1 Table — (DOCX) [file pone.0135911.s006.docx]

| Gene: |  | **Ruffe:** | **Flounder:** |
| --- | --- | --- | --- |
| ApoIA | for | GTGTGCTTGGCGGAATACTC | CTGTGTATGTCTTCGCTTGTG |
|  | rev | GTGACCTCCGTACCTCTATC | CTTCACATGCACACACAGAAC |
| Amplicon [bp] | | 155 | 154 |
| Ca | for | AGTTGTCCGCCATGCAGCAT |  |
|  | rev | GACTCTTCTTCCTGGTTCTCT |  |
| Amplicon [bp] | | 188 |  |
| Eno-alpha | for | CGACTTGGCTGGAAACACAG | GGAGCATCAACTGGAATCTAC |
|  | rev | TTCAGCGTGTGGTACAGCTC | CCGTCCATGTCAATCATCATC |
| Amplicon [bp] | | 174 | 185 |
| Hsp70 | for |  | ATGCAGTCATCACAGTTCCTG |
|  | rev |  | GATGAGGACATTACGTTCACTG |
| Amplicon [bp] | |  | 170 |
| TIM | for | CGTATGTTCTCCTGCCATTAG |  |
|  | rev | TCTGGCTAGACATCCTATCTATT |  |
| Amplicon [bp] | | 123 |  |
| Actb | for |  | CTTCTACAACGAGCTGAGAGT |
|  | rev |  | TGTGATCTCCTTCTGCATCCT |
| Amplicon [bp] | |  | 197 |

S1 Table. Primer sequences used for the qRT-PCR experiments and the amplicon size of each of the analysed gene.
