# Supplementary material for: Evaluating the Hypoxia Response of Ruffe and Flounder Gills by a Combined Proteome and Transcriptome Approach
Source: PLoS One. 2015 Aug 14;10(8):e0135911. doi: 10.1371/journal.pone.0135911 (PMC4537130; doi:10.1371/journal.pone.0135911)
Supplement: S2 Table — The table shows the results of the reciprocal blastn analyses with an e-value cut-off of 1e-10. (DOC) [file pone.0135911.s007.doc]

S2 Table. Comparison of ruffe and European flounder transcriptomes.

|  | Ruffe (*G. cernua*) | Flounder (*P. flesus*) |
| --- | --- | --- |
| Contigs ≥500 bp | 34,573 | 29,991 |
| Blastn ≤1E-10 | 30,742 | 29,062 |
| Reciprocal contig pairs | 17,928 | |
| Unique hits | 12,774 | 11,110 |
| No hits | 40 | 24 |
| Blastn approach | Ruffe contigs vs flounder database | Flounder contigs vs ruffe database |
|

The table shows the results of the reciprocal blastn search with an e-value cut-off of 1e-10.
