# Supplementary material for: Evaluating the Hypoxia Response of Ruffe and Flounder Gills by a Combined Proteome and Transcriptome Approach
Source: PLoS One. 2015 Aug 14;10(8):e0135911. doi: 10.1371/journal.pone.0135911 (PMC4537130; doi:10.1371/journal.pone.0135911)
Supplement: S3 Table — The GO terms were derived from the blastx annotated contigs of ruffe and flounder against the human database. (DOC) [file pone.0135911.s008.doc]

**S3 Table. PANTHER analysis of ruffe and flounder transcriptomes. The GO terms were derived from the blastx annotated contigs of ruffe and flounder against the human database.**

|  |  | Ruffe |  | Flounder |  |
| --- | --- | --- | --- | --- | --- |
|  |  | No. | % | No. | % |
| molecular function | |  |  |  |  |
| 1 | antioxidant activity (GO:0016209) | 17 | 0.20% | 16 | 0.20% |
| 2 | translation regulator activity (GO:0045182) | 85 | 0.90% | 82 | 0.90% |
| 3 | protein binding transcription factor activity (GO:0000988) | 94 | 1.00% | 93 | 1.00% |
| 4 | enzyme regulator activity (GO:0030234) | 536 | 5.80% | 549 | 6.00% |
| 5 | transporter activity (GO:0005215) | 615 | 6.70% | 571 | 6.30% |
| 6 | structural molecule activity (GO:0005198) | 626 | 6.80% | 621 | 6.80% |
| 7 | receptor activity (GO:0004872) | 715 | 7.80% | 689 | 7.60% |
| 8 | nucleic acid binding transcription factor activity (GO:0001071) | 738 | 8.00% | 731 | 8.00% |
| 9 | binding (GO:0005488) | 2830 | 30.70% | 2818 | 30.90% |
| 10 | catalytic activity (GO:0003824) | 3020 | 32.80% | 3057 | 33.50% |
| biological process | |  |  |  |  |
| 1 | growth (GO:0040007) | 4 | 0.00% | 1 | 0.00% |
| 2 | locomotion (GO:0040011) | 6 | 0.10% | 5 | 0.10% |
| 3 | reproduction (GO:0000003) | 231 | 2.50% | 231 | 2.50% |
| 4 | apoptotic process (GO:0006915) | 343 | 3.70% | 334 | 3.70% |
| 5 | biological adhesion (GO:0022610) | 467 | 5.10% | 468 | 5.10% |
| 6 | cellular component organization or biogenesis (GO:0071840) | 627 | 6.80% | 630 | 6.90% |
| 7 | response to stimulus (GO:0050896) | 697 | 7.60% | 699 | 7.70% |
| 8 | immune system process (GO:0002376) | 715 | 7.80% | 734 | 8.10% |
| 9 | multicellular organismal process (GO:0032501) | 884 | 9.60% | 886 | 9.70% |
| 10 | localization (GO:0051179) | 1398 | 15.20% | 1387 | 15.20% |
| 11 | developmental process (GO:0032502) | 1451 | 15.80% | 1389 | 15.20% |
| 12 | biological regulation (GO:0065007) | 1476 | 16.00% | 1493 | 16.40% |
| 13 | cellular process (GO:0009987) | 2969 | 32.20% | 2949 | 32.40% |
| 14 | metabolic process (GO:0008152) | 4470 | 48.50% | 4469 | 49.00% |
| cellular component | |  |  |  |  |
| 1 | synapse (GO:0045202) | 5 | 0.10% | 5 | 0.10% |
| 2 | cell junction (GO:0030054) | 44 | 0.50% | 42 | 0.50% |
| 3 | extracellular matrix (GO:0031012) | 170 | 1.80% | 197 | 2.20% |
| 4 | membrane (GO:0016020) | 236 | 2.60% | 229 | 2.50% |
| 5 | extracellular region (GO:0005576) | 240 | 2.60% | 267 | 2.90% |
| 6 | macromolecular complex (GO:0032991) | 336 | 3.60% | 305 | 3.30% |
| 7 | organelle (GO:0043226) | 545 | 5.90% | 536 | 5.90% |
| 8 | cell part (GO:0044464) | 869 | 9.40% | 850 | 9.30% |
