# Supplementary material for: Evaluating the Hypoxia Response of Ruffe and Flounder Gills by a Combined Proteome and Transcriptome Approach
Source: PLoS One. 2015 Aug 14;10(8):e0135911. doi: 10.1371/journal.pone.0135911 (PMC4537130; doi:10.1371/journal.pone.0135911)
Supplement: S4 Table — (DOC) [file pone.0135911.s009.doc]

**S4 Table.** Proteins that changed under hypoxia in the ruffe gills (cf. Fig. 3A).

| spot | mean normoxia | SD normoxia | mean hypoxia | SD hypoxia |
| --- | --- | --- | --- | --- |
| 9 | 7.19687 | 4.140854643 | 24.94204 | 3.45750059 |
| 10 | 10.23814 | 1.804878712 | 30.96891 | 7.399459543 |
| 12 | 6.1506 | 2.449730114 | 39.56755 | 11.07987567 |
| 13 | 75.15213 | 26.26237976 | 5.80001 | 4.098232386 |
| 14 | 40.14496 | 17.10266738 | 3.01593 | 2.044208735 |
| 18 | 36.56952 | 8.511341604 | 19.86127 | 0.59759544 |
| 20 | 9.47255 | 1.734031849 | 3.36998 | 0.26539893 |
| 25 | 12.56036 | 5.159642673 | 3.82722 | 2.083191501 |
| 37 | 8.96821 | 2.675675674 | 4.10053 | 1.07390537 |
| 44 | 37.49644 | 1.849777414 | 14.33525 | 6.864593729 |
| 52 | 13.38182 | 3.128792856 | 51.83032 | 15.00669374 |
| 57 | 160.58515 | 1.029762576 | 143.66794 | 8.418938702 |
| 61 | 33.34138 | 9.872544598 | 10.91187 | 6.244500729 |
| 82 | 123.15404 | 13.0914371 | 23.31383 | 3.540645634 |
| 83 | 45.93537 | 2.026584573 | 80.7936 | 16.95492525 |
| 84 | 45.1685 | 4.093580826 | 20.85304 | 9.744454066 |
| 85 | 17.45937 | 1.096631164 | 38.98996 | 6.069498725 |
| 89 | 17.12722 | 2.720786772 | 23.66511 | 2.093626074 |
| 90 | 8.93487 | 2.529852888 | 15.60404 | 1.177604524 |
| 91 | 23.85899 | 2.132982442 | 34.70321 | 5.696450211 |
| 92 | 10.82478 | 0.951371772 | 21.32213 | 3.569231554 |
| 97 | 29.0741 | 0.672084718 | 9.24776 | 3.020386794 |
| 99 | 17.36977 | 1.533442736 | 25.30496 | 2.709084061 |
| 102 | 157.61441 | 34.09475591 | 49.61173 | 31.27508809 |
| 106 | 18.91669 | 5.621815141 | 35.12094 | 3.401989036 |
| 107 | 8.69143 | 2.842105189 | 29.53756 | 12.0022897 |
| 117 | 15.29119 | 5.342813746 | 4.01426 | 2.23564364 |
| 133 | 8.79377 | 1.892631005 | 46.9976 | 23.46753274 |
| 134 | 40.43719 | 4.456336443 | 9.74118 | 1.939621562 |
| 137 | 29.7283 | 10.05271662 | 57.9846 | 15.70474486 |
| 147 | 2.64694 | 0.763836169 | 5.91098 | 1.843068215 |
| 149 | 2.03427 | 1.686504647 | 8.58998 | 2.84473486 |
