# Supplementary material for: Evaluating the Hypoxia Response of Ruffe and Flounder Gills by a Combined Proteome and Transcriptome Approach
Source: PLoS One. 2015 Aug 14;10(8):e0135911. doi: 10.1371/journal.pone.0135911 (PMC4537130; doi:10.1371/journal.pone.0135911)
Supplement: S5 Table — (DOC) [file pone.0135911.s010.doc]

**S5 Table.** Proteins that changed under hypoxia in the flounder gills (cf. Fig. 3B).

| spot | mean normoxia | SD normoxia | mean hypoxia | SD hypoxia |
| --- | --- | --- | --- | --- |
| 10 | 31.07829 | 5.364152101 | 54.29104 | 10.2327805 |
| 17 | 63.41184 | 29.64771022 | 3.18659 | 1.76199762 |
| 19 | 19.22886 | 4.579689489 | 6.16553 | 3.30699261 |
| 23 | 12.41964 | 1.241773451 | 3.65553 | 2.35703396 |
| 24 | 14.6106 | 0.889128765 | 6.54716 | 4.14360194 |
| 30 | 95.73242 | 7.127571197 | 73.10701 | 8.34484605 |
| 32 | 9.91196 | 3.348536553 | 22.19139 | 5.46097057 |
| 33 | 116.43081 | 17.01107802 | 83.71324 | 0.71747248 |
| 40 | 1.19404 | 0.903871099 | 10.44854 | 5.78179066 |
| 43 | 39.37789 | 12.31108588 | 79.64883 | 3.5732715 |
| 52 | 177.75394 | 54.42283356 | 308.3135 | 12.9109932 |
| 54 | 12.92336 | 1.072875186 | 9.8306 | 1.16866605 |
| 56 | 10.78349 | 1.869842141 | 27.94649 | 0.91861192 |
| 61 | 42.11747 | 1.517337692 | 13.04493 | 4.03281619 |
| 70 | 10.00444 | 2.366207261 | 1.09121 | 0.6218854 |
| 71 | 9.44727 | 1.197297059 | 0.44348 | 0.16317505 |
| 80 | 15.66573 | 2.007644724 | 2.89353 | 2.20621401 |
| 82 | 35.27493 | 3.157871333 | 4.80232 | 4.13724031 |
| 83 | 9.51044 | 3.849194937 | 1.23462 | 0.96429329 |
| 87 | 172.19888 | 14.1594999 | 51.71991 | 14.1035126 |
| 89 | 9.01741 | 3.524419887 | 1.46329 | 0.56177316 |
| 90 | 135.74446 | 57.0730202 | 46.15263 | 14.8306156 |
| 93 | 154.43062 | 28.25896119 | 56.02624 | 3.64625838 |
| 96 | 12.16323 | 1.283739053 | 6.03241 | 2.6866645 |
| 99 | 34.5 | 27.7 | 142.6 | 71.2 |
| 105 | 46.48701 | 10.42273249 | 14.45784 | 6.51642265 |
| 114 | 13.86876 | 2.399409885 | 2.75343 | 1.39523365 |
| 115 | 203.27372 | 6.190219269 | 104.66234 | 0.62036679 |
| 116 | 33.10631 | 5.478546115 | 15.08282 | 0.82698322 |
| 117 | 6.33576 | 0.82810042 | 4.08353 | 0.89811629 |
| 119 | 5.50006 | 0.248278711 | 3.41101 | 0.98336153 |
