# Supplementary material for: Evaluating the Hypoxia Response of Ruffe and Flounder Gills by a Combined Proteome and Transcriptome Approach
Source: PLoS One. 2015 Aug 14;10(8):e0135911. doi: 10.1371/journal.pone.0135911 (PMC4537130; doi:10.1371/journal.pone.0135911)
Supplement: S6 Table — (DOC) [file pone.0135911.s011.doc]

**S6 Table.** Protein identifications and abundance ratios in response to hypoxia in the gills of the ruffe. Note that the log2 transformed *p*-values have not been corrected for multiple comparison.

| **Regulation**  **(ratio)**  Up Down | **Identification** | **Protein ID** | **Spot**  **ID** | **Mr (kDa)** | **pI** | **Gel Mr (kDa)** | **Gel pI** | **Mean: Normoxia/ Hypoxia** | **p-value**  **(T-test; Bonferroni)** | **p-value**  **(log2)** | **Function** | **Identified in UniProtKB** | **Identified in transcriptome** | **Accession number** |
| --- | --- | --- | --- | --- | --- | --- | --- | --- | --- | --- | --- | --- | --- | --- |
| *Reference* | glyceraldehyde-3-phosphate dehydrogenase | GAPDH | 1 | 35.8 | 6.4 | 33 | 6.6 |  |  |  | energy metabolism | yes | yes | AEM60004 |
| 5.3 | hemoglobin beta | Hbβ | 133 | 16.4 | 7.0 | 12 | 6.75 | 8.8± 1.9 46.9 ± 23.5 | 4.0 x 10^-5^ | 0.0834 | respiratory protein | yes | no | AAO27764 |
| 4.2 | enolase β | Enoβ | 149 | 47.5 | 6.3 | 49 | 6.6 | 2.0± 1.7 8.6 ± 2.8 | 0.042 | 0.0486 | energy metabolism | yes | yes | ADG29136 |
| 2.9 | 6-phosphogluconat-dehydrogenase | Pgd | 52 | 53.2 | 6.2 | 44 | 6.5 | 13.4± 3.1 51.8 ± 15.0 | 3.9 x 10^-5^ | 0.0239 | energy metabolism | no | yes | XP_003963174 |
| 2.2 | enolase α | Enoα | 147 | 47.1 | 6.1 | 51 | 6.0 | 2.6± 0.8 5.9± 1.8 | 0.016 | 0.0817 | energy metabolism | yes | yes | AAH71359 |
| 2.2 | triosephosphat isomerase | TIM | 85 | 26.7 | 6.9 | 26 | 6.45 | 17.5± 1.1 38.9± 6.1 | 0.0004 | 0.0078 | energy metabolism | yes | yes | XP_004550861 |
| 1.8 | carbonic anhydrase | CA | 90 | 28.5 | 6.7 | 25 | 6.25 | 8.9± 2.5 15.6± 1.2 | 0.039 | 0.0278 | acid-base homeostasis | no | yes | ACU30151 |
| 1.9 | phoshoglycerate mutase | Pgm | 92 | 28.9 | 6.0 | 26 | 6.35 | 10.8± 1.0 21.3± 3.6 | 0.007 | 0.0159 | energy metabolism | no | yes | XP_004539107 |
| 1.86 | immunglobulin light chain | Igl2 | 106 | 25.9 | 5.4 | 26 | 5.15 | 18.9± 5.6 35.1± 3.4 | 0.0012 | 0.0252 | immune response | no | no | AAS55942 |
| 1.46 | immunglobulin light chain | Igl1 | 99 | 25.9 | 5.4 | 25 | 6.2 | 17.4± 1.5 25.3± 2.7 | 0.02 | 0.0227 | immune response | yes | yes | AAS55942 |
| 0.54 | heat shock protein 70 | Hsp70 | 18 | 70.2 | 5.3 | 68 | 5.0 | 36.6± 8.5 19.9± 0.6 | 0.001 | 0.0504 | molecular chaperone | yes | yes | NP_001266600 |
| 0.38 | rab GTPase-binding effector protein 2-like | Rabep | 44 | 52.5 | 6.1 | 52 | 5.9 | 37.5± 1.9 14.3± 6.9 | 0.0003 | 0.01 | cell signalling | no | yes | XP_004560719 |
| 0.32 | apolipoprotein | Apo I | 102 | 29.0 | 5.3 | 26 | 4.85 | 157.6± 34.1 49.6± 31.2 | 6.3 x 10^-7^ | 0.0299 | lipid metabolism | yes | yes | ACF21981 |
| 0.19 | 14-3-3-protein | 14-3-3 | 82 | 27.8 | 4.6 | 29 | 4.55 | 123.2± 13.1 23.3± 3.5 | 8.6 x 10^-7^ | 0.0005 | cell signalling | yes | yes | XP_004070571 |
