# Supplementary material for: Evaluating the Hypoxia Response of Ruffe and Flounder Gills by a Combined Proteome and Transcriptome Approach
Source: PLoS One. 2015 Aug 14;10(8):e0135911. doi: 10.1371/journal.pone.0135911 (PMC4537130; doi:10.1371/journal.pone.0135911)
Supplement: S7 Table — (DOC) [file pone.0135911.s012.doc]

**S7 Table.** Protein identifications and abundance ratios in response to hypoxia in the gills of the flounder. Note that the log2 transformed *p*-values have not been corrected for multiple comparison.

| **Regulation**  **(ratio)**  Up Down | **Protein name** | **Protein ID** | **Spot**  **ID** | **Mr (kDa)** | **pI** | **Gel Mr (kDa)** | **Gel pI** | **Mean: Normoxia/ Hypoxia** | **p-value**  **(T-test; Bonferroni)** | **p-value**  **(log2)** | **Function** | **Identified in UniProtKB** | **Identified in transcriptome** | **Accession number** |
| --- | --- | --- | --- | --- | --- | --- | --- | --- | --- | --- | --- | --- | --- | --- |
| *Reference* | glyceraldehyde-3-phosphate dehydrogenase | GAPDH | 2 | 36 | 6.2 | 48 | 5.65 |  |  |  | energy metabolism | no | yes | ACF35053 |
| 2.59 | filamin B-like | Flnb | 56 | 47.5 | 5.3 | 45 | 5.1 | 10.8± 1.9 27.0± 0.9 | 0.0001 | 0.0003 | cytoskeleton | yes | yes | XP_005453685 |
| 2.24 | Willebrand factor A domain-containing protein 5A-like | vWF5A | 32 | 67.6 | 6.16 | 68 | 5.2 | 9.9± 3.35 22.2± 5.5 | 0.0019 | 0.0535 | hemostasis | yes | yes | XP_004075971 |
| 2.02 | enolase α | Enoα | 43 | 47 | 6.1 | 48 | 5.2 | 39.4±12.8  9.7± 3.6 | 1.7 x 10^-5^ | 0.089 | energy metabolism | yes | yes | NP_997887 |
| 0.65 | heat shock protein 70 | Hsp70 | 30 | 71.1 | 5.3 | 69 | 5.5 | 95.7± 7.1 73.1± 8.3 | 0.0002 | 0.0434 | molecular chaperone | yes | yes | AAC33859 |
| 0.52 | transferrin 2 | Tf2 | 115 | 74.7 | 6.1 | 70 | 5.6 | 203.3± 6.2 104.7± 0.6 | 4.8 x 10^-7^ | 0.0002 | ferric iron binding | yes | no | AAF33233 |
| 0.5 | proteasome subunit  β type-2 | PSM β2 | 96 | 22.5 | 6.0 | 26 | 5.85 | 12.2± 1.3 6.0± 2.7 | 0.0.0291 | 0.0436 | protein degradation | yes | yes | ACO09331 |
| 0.46 | transferrin 1 | Tf1 | 116 | 74.7 | 6.1 | 70 | 5.7 | 33.1± 5.5 15.1± 0.8 | 0.0004 | 0.01 | ferric iron binding | yes | no | AAF33233 |
| 0.36 | apolipoprotein | Apo III | 93 | 28.7 | 5.6 | 25 | 5.3 | 154.3± 28.3 56.0± 3.6 | 4.9 x 10^-7^ | 0.0081 | lipid metabolism | yes | yes | ADV03093 |
| 0.34 | apolipoprotein | Apo II | 90 | 28.7 | 5.6 | 25 | 5.25 | 135.7± 57.1 48.2± 14.8 | 7.1 x 10^-7^ | 0.0981 | lipid metabolism | yes | yes | ADV03093 |
| 0.31 | actin β | β-Act | 61 | 41.7 | 5.3 | 38 | 5.55 | 42.1± 1.5 13.0± 4.0 | 0.0002 | 0.0007 | cytoskeleton | yes | yes | AAF63665 |
| 0.3 | apolipoprotein | Apo I | 87 | 28.7 | 5.6 | 25 | 5.25 | 172.2± 14.1 51.7±4.8 | 2.1 x 10^-7^ | 0.001 | lipid metabolism | yes | yes | ADV03093 |
| 0.19 | periplakin | Ppl | 80 | 201 | 5.8 | 29 | 5.95 | 15.7± 2.0 2.9± 2.2 | 0.0017 | 0.0038 | cytoskeleton | no | no | XM_005462862 |
| 0.14 | proteasome subunit  α type-6 | PSM α6 | 82 | 27.3 | 6.4 | 28 | 6.0 | 35.3± 3.1 4.8± 4.1 | 0.0001 | 0.0012 | protein de**grad**ation | yes | no | ACQ58552 |
